# Loss of PDE4D7 expression promotes androgen independence, neuroendocrine differentiation and alterations in DNA repair: implications for therapeutic strategies

**DOI:** 10.1038/s41416-023-02417-5

**Published:** 2023-09-22

**Authors:** Chloe Gulliver, Sebastian Huss, Axel Semjonow, George S. Baillie, Ralf Hoffmann

**Affiliations:** 1 School of Cardiovascular & Metabolic Health, University of Glasgow, Glasgow, G12 8TA Scotland UK; 2 Gerhard-Domagk-Institute of Pathology, University Hospital Münster, 48149 Münster, Germany; 3 Prostate Center, University Hospital Münster, 48149 Münster, Germany; 4 Oncology Solutions, Philips Research Europe, High Tech Campus 34, 5656AE Eindhoven, The Netherlands

**Keywords:** Prostate cancer, Cancer genomics, Tumour biomarkers

## Abstract

**Background:**

Androgen signalling remains the seminal therapeutic approach for the management of advanced prostate cancer. However, most tumours eventually shift towards an aggressive phenotype, characterised by androgen independence and treatment resistance. The cyclic adenosine monophosphate (cAMP) pathway plays a crucial role in regulating various cellular processes, with the phosphodiesterase PDE4D7 being a vital modulator of cAMP signalling in prostate cancer cells.

**Methods:**

Using shRNA-mediated PDE4D7 knockdown in LNCaP cells and downstream analysis via RNA sequencing and phenotypic assays, we replicate clinical observations that diminished PDE4D7 expression promotes an aggressive prostate cancer phenotype.

**Results:**

Our study provides evidence that loss of PDE4D7 expression represents a pivotal switch driving the transition from an androgen-sensitive state to hormone unresponsiveness and neuroendocrine differentiation. In addition, we demonstrate that PDE4D7 loss affects DNA repair pathways, conferring resistance to poly ADP ribose polymerase (PARP) inhibitors.

**Conclusion:**

Reinstating PDE4D7 expression sensitises prostate cancer cells to anti-androgens, DNA damage response inhibitors, and cytotoxic therapies. These findings provide significant insight into the regulatory role of PDE4D7 in the development of lethal prostate cancer and the potential of its modulation as a novel therapeutic strategy.

## Introduction

In 2020, ~1.4 million men were newly diagnosed with prostate cancer (PCa) worldwide, leading to 375,000 cancer-induced deaths [1]. In general, the majority of PCa patients are at low-to-intermediate risk of developing progressive disease [2], however, 17–31% of patients are clinically high-risk with an elevated probability of PCa-specific death [3, 4].

In 2012, the U.S. Preventive Services Task Force (USPSTF) recommended against continuously widespread PSA screening in the U.S. to avoid overdetection of low-risk diseases [5]. In following years, the number of newly diagnosed patients declined across all age groups by an estimated annual percent change of nearly 20% [6], and the incidence of patients with locally advanced or metastatic disease at presentation increased [7, 8]. This in turn may promote rising PCa mortality [9].

Despite the shift to a higher grade and advanced stage at diagnosis, the identified tumours are still heterogeneous concerning their biological and progressive characteristics. The most frequent molecular alterations in advanced PCa involve signalling via androgen receptor (AR), WNT, PI3K, DNA repair, and the cell cycle [10, 11], as well as altered gene expression and epigenetics [12, 13]. Consequently, it is clinically relevant to accurately risk stratify patients to apply the most appropriate therapeutic strategy [14].

Cyclic AMP (cAMP) can modulate AR activity via promoting phosphorylation of the AR [15] or AR coregulatory proteins [16] by protein kinase A (PKA). Alternatively, cAMP can activate cAMP response element-binding protein (CREB) through PKA phosphorylation in the presence of androgens and thus bind to promoter regions of androgen-regulated genes to enhance their transcriptional activation [17]. Phosphodiesterases (PDEs) are a superfamily of enzymes [18] that play a fundamental role in hydrolysing cAMP, leading to its degradation and termination of cell signal transduction in a spatially compartmentalised manner [19].

Previously, we reported that the expression of PDE4D7 is increased in TMPRSS2-ERG-positive prostate tumours [20] and is inversely correlated with the risk of biochemical recurrence (BCR) after radical prostatectomy (RP). The PDE4D7 score independently adds value to clinical prognostic variables such as ISUP Gleason grade or the CAPRA/CAPRA-S risk scores to predict postsurgical disease progression and poor prognosis [21, 22]. Furthermore, we showed that PDE4D7 transcription is associated with androgen resistance and might be involved in the regulation of PCa cell proliferation [23].

Here, we present evidence that the PDE4D7 score predicts survival outcomes in clinically high-risk PCa patients after postsurgical PSA recurrence followed by salvage radiation therapy (SRT) with or without androgen-deprivation therapy (ADT). We replicate in a cellular model the clinical observation that diminished PDE4D7 expression promotes an aggressive phenotype and induces therapeutic resistance, and we suggest the manipulation of PDE4D7 expression or activity as a novel therapeutic avenue.

## Methods

### Patient samples

Two biopsy punches (~1 ×;2 mm) were collected from 367 PCa patients operated on between 1994 and 2011. Of those, 363 patients had at least one adverse pathological feature (PSM: positive surgical margins; SVI: seminal vesicle invasion; EPE: extra-prostatic extension; LNI: lymph node invasion; Gleason 4 component) and/or were classified into post-treatment intermediate- or high risk of disease progression according to the clinical risk metrics CAPRA-S [24] and/or the EAU-BCR risk model [25], while four patients demonstrated a post-operatively rising PSA in the absence of any of the stated adverse features. All patients experienced BCR and were stratified to SRT. In total, 188 patients also received ADT (Supplementary Fig. 1). The study was conducted in accordance with the Declaration of Helsinki and approved by the institutional Ethics Committee of Westfalian Wilhelms University Münster, Germany (Ethics code 2007-467-f-S). Informed consent was obtained from all subjects involved in this study.

### Generation of stable PDE4D7-modified LNCaP cells

#### Stable shRNA-mediated PDE4D7 knockdown in LNCaP cells

LNCaP clone FGC (ATCC® CRL1740™) cells were cultured in RPMI 1640 medium (Gibco™) supplemented with 10% (v/v) foetal bovine serum, 1% (v/v) l-glutamine and 1% (v/v) penicillin/streptomycin. The lentiviral transfer plasmid was designed to stably express an shRNA hairpin targeting the first coding exon of the PDE4D7 transcript. As a control, a scrambled nucleotide sequence of the PDE4D7-targeting shRNA was cloned into the transfer plasmid. LNCaP FGC cells were infected with lentiviruses at MOI = 10 in the presence of 10 µg/ml polybrene. Cells were maintained in a puromycin-supplemented medium prior to selection and clonal expansion of stably transduced cells (Supplementary Materials).

#### Inducible PDE4D7 expression in PDE4D7-knockdown LNCaP P1 cells

The PDE4D7 (GenBank ID AF536976) sequence was first subcloned into lentivirus vectors with CMV (LVR-1001-pLV-CMV-PGK-Puro) and then into the Tet-On inducible vector (pLV-tet-on-3G-P2A-Puro).

LNCaP-shRNA P1 cells were cultured in the same conditions to LNCaP FGC cells, and infected with Tet-On inducible lentivirus at MOI = 5–10 in the presence of 8 µg/ml polybrene (Supplementary Materials). PDE4D7 expression was induced with 1 µg/ml doxycycline.

For downstream molecular analysis, the following cell lines/clones were used: LNCaP FCG wild type (WT); LNCaP scrambled shRNA control (clone SC2); LNCaP PDE4D7-targeting shRNA (clones P1, w5.2 and w6.3), and LNCaP clone P1 transfected with Tet-On inducible PDE4D7 vector (clone P1-TET4D7).

### RT-qPCR

To account for potential tumour heterogeneity, the two tissue punches of the RP cohort were combined before RNA extraction as previously described [21]. RNA was extracted from LNCaP cell lines using the RNeasy Mini Kit (QIAGEN). RT-qPCR, oligonucleotide primers/probes (Supplementary Table 7) and statistical analysis were performed as previously described [21]. Details on the calculation of clinical and genomic risk scores can be found in Supplementary Materials.

### RNA sequencing

Total RNA (100 ng) was used as input to remove ribosomal RNA using the Ribo-Zero Gold (Human/Mouse/Rat) rRNA Removal Kit (Illumina Inc.) according to the manufacturer’s instructions. The total of the depleted RNA was used as input into the Scriptseq V2 RNA-Seq Library Preparation Kit (Epicentre/Illumina Inc.). Prepared RNAseq libraries were sequenced using a NextSeq 500 sequencing system (paired-end; 2 × 75 bp read length; ~80 million total reads per sample for human tissue and ~20 million reads per sample for the sequenced cell lines). Details on RNAseq data processing can be found in the Supplementary Materials.

### Gene set enrichment analysis

Gene set enrichment analysis (GSEA) was performed as described here (https://www.gsea-msigdb.org/gsea/index.jsp). The feature count matrices of the gene expression derived from RNAseq were used as GSEA input.

### Whole-genome sequencing

Genomic DNA (100 ng) was fragmented at 350 bp by ultrasonication. Libraries were constructed following the TruSeq DNA Nano protocol (Illumina). In brief, sheared gDNA was end-prepped, dA-tailed and enriched for fragments of ~350 bp through size selection with AMPure XP beads (Beckman Coulter). Indexing PCR for a total of eight cycles was performed for the size-selected fragments, and the products were purified with AMPure XP beads. The quality of the final libraries was checked on the LabChip GX Nucleic Acid Analyzer (PerkinElmer). High-coverage sequencing was performed on the Illumina NovaSeq 6000 system with the S4 flow cell and PE150 configuration. Further details can be found in the Supplementary Materials.

### Plasmid DNA transfection

LNCaP cells were transfected with pcDNA3.1-PDE4D7-VSV plasmid DNA using Lipofectamine LTX with Plus Reagent (Thermo Fisher Scientific) according to the manufacturer’s protocol.

### xCELLigence real-time cell analysis

Real-time cell growth was analysed using the xCELLigence RTCA system (Agilent). Cellular adhesion influences electrical impedance, which is converted into the cell index (CI) via RTCA software. Cells were treated ~24 h post-seeding (10,000 cells/well). Negative controls (0.1% DMSO or untreated) were also included. CI was transformed into normalised CI to timepoint of treatment.

### Western blotting

Cells were lysed in 3T3 buffer (50 mM NaCl, 50 mM NaF, 30 mM sodium pyrophosphate, 25 mM HEPES, 2.5 mM EDTA, 10% (v/v) glycerol, 1% (v/v) Triton X-100; pH 7.5). Equal protein concentrations were separated via SDS-PAGE and transferred onto nitrocellulose membranes. Membranes were blocked in 5% (w/v) milk powder in TBS-T for 1 h prior to primary and secondary antibody (Supplementary Materials) incubations and imaged via Odyssey DLx (LICOR).

### Immunofluorescence

Cells were seeded onto 13-mm glass coverslips. Twenty-four hours post-transfection coverslips were fixed with 4% (v/v) paraformaldehyde, prior to blocking and permeabilization (10% donkey serum, 0.5% BSA and 0.2% Triton-X in PBS). Coverslips were incubated overnight with anti-PDE4D7 primary (1:200, Baillie Lab) and Alexa-Fluor 488 Donkey-anti-Goat secondary (1:500, Thermo Fisher Scientific #A-11055), or anti-γH2AX primary (1:500, Sigma-Aldrich #05-636) and Alexa-Fluor 488 Donkey-anti-Mouse secondary (1:500, Thermo Fisher Scientific #A-21200) antibodies. Coverslips were mounted with Duolink In Situ Mounting Medium with DAPI (Sigma-Aldrich) and imaged via ZEISS LSM 880 laser scanning microscope with a ×63 oil immersion objective.

### Statistical analysis

MedCalc (MedCalc Software v2.20 BVBA, Ostend, Belgium) was used for statistical analysis of patient sample data. For LNCaP data normality was assessed via QQ plot, and all data are expressed as mean +/− standard error of the mean (SEM). For statistical analysis of three or more groups with one independent variable, a one-way ANOVA with Dunnet post hoc test or Welch and Brown–Forsythe ANOVA (for data with significant standard deviation) were used. A two-way ANOVA with the Sidak post hoc test was used for analysis of two independent variables. Comparison between the two groups was performed via unpaired Student’s *t* test. Statistical analyses were performed via GraphPad Prism Version 9 (*P ≤ 0.05, **P ≤ 0.01, ***P ≤ 0.001, ****P ≤ 0.0001).

## Results

### The PDE4D7 score is associated with survival outcomes in high-risk patients

All subjects enrolled in this study experienced postsurgical PSA relapse and were subsequently stratified to SRT. Pathological analysis revealed pT3a or higher in nearly 80% of patients (Table 1). Most patients (85%) were classified as intermediate-risk (41.1%; CAPRA-S scores 2–5) or high-risk (44.1%; CAPRA-S scores >5) for postsurgical disease progression [24]. Over half of the patients (188/367) received ADT during the study period. During a median follow-up of 103 months post prostatectomy, 11.7% died from prostate cancer-specific mortality (PCSM) and 17.4% from all-cause mortality (ACM) (Table 1).

**Table 1:** Aggregated summary of the characteristics of the studied patient cohort.

# Patients	*N* = 367	
Age range [years]	45.3–79.2 (median: 62.5; IQR: 8.1)	
preoperative PSA [ng/ml]	0.3–168 (median: 10.0; IQR: 9.3)	
pT stage	*N*	%
pT2	78	21.3%
pT3a	131	35.7%
pT3b	103	28.1%
pT3c	44	12.0%
pT4	11	3.0%
ISUP Gleason		
Grade Group 1	36	9.8%
Grade Group 2	147	40.1%
Grade Group 3	109	29.7%
Grade Group 4	19	5.2%
Grade Group 5	55	15.0%
Not available	1	0.3%
CAPRA-S score		
CAPRA-S < = 2	39	10.6%
CAPRA-S 3–5	151	41.1%
CAPRA-S > = 6	162	44.1%
Not available	15	4.1%
EAU-BCR risk score		
EAU-BCR Risk 0	185	50.4%
EAU-BCR Risk 1	163	44.4%
Not available	19	5.2%
Surgical margin status		
Negative	100	27.2%
Positive	265	72.2%
Not available	2	0.5%
Extra-prostatic extension		
Absent	135	36.8%
Present	229	62.4%
Not available	3	0.8%
Seminal vesicle invasion		
Absent	244	66.5%
Present	123	33.5%
Lymph node invasion		
Absent	334	91.0%
Present	33	9.0%
Clinical metastasis		
No event	182	49.6%
Event	128	34.9%
Unknown	57	15.5%
Salvage radiation		
Not administered	0	0%
Administered	367	100%
Androgen-deprivation therapy		
Not administered	162	44.1%
Administered	188	51.2%
Unknown	17	4.6%
Prostate cancer-specific mortality		
No event	324	88.3%
Event	43	11.7%
All-cause mortality		
No event	303	82.6%
Event	64	17.4%
Median Follow-Up [post-RP]		
	103 months	

*ADT* androgen-deprivation therapy, *CR* clinical metastases, *PCSM* prostate cancer-specific mortality, *ACM* all-cause mortality, *N/A* not available, *RP* radical prostatectomy, *SRT* salvage radiation therapy.

Demographics of the RP patient cohort including the *N* = 367 patients eligible for statistical data analysis. For patient age and preoperative PSA, the min and max values in the cohort are shown; median and IQR are shown in parentheses. Postsurgical pathology is given (note: extracapsular extension was derived from pathology-stage information). The outcome category illustrates the cumulative events in terms of recurrence to CR, SRT or ADT after surgery. Mortality is shown as PCSM as well as overall ACM; N/A.

We observed a strong inverse association between the PDE4D7 score and PCSM after SRT in multivariable analysis (HR = 0.37; 95% CI 0.23–0.58; *P* < 0.0001) (Table 2). We then sought to determine whether the PDE4D7 score provided independent prognostic information in the context of genomic GPS (Genomic Prostate Score [26]) or CCP (Cell Cycle Progression [26]) signatures (see also Supplementary Materials for details on the calculation of the respective score). Interestingly, only the Genomic Prostate Score signature demonstrated a significant association with PCSM (HR = 1.3; 95% CI 1.0–1.6; *P* = 0.03). In both analyses, the PDE4D7 score remained a significant variable (*P* = 0.01 and *P* = 0.007; Supplementary Table 1A and 1B, respectively). Additionally, the PDE4D7 score was tested in in combination with the CAPRA-S and EAU-BCR risk scores [24, 25], and remained the most significant predictor for PCSM after SRT in both models (HR = 0.36, *P* < 0.0001; HR = 0.31, *P* < 0.0001, respectively; Supplementary Table 1C).

**Table 2:** Multivariable Cox regression analysis of the PDE4D7 score with the clinical prognostic parameters in a *N* = 342 clinical high-risk prostate cancer patient cohort.

*N* = 342 patients	Multivariable		
Variable	HR	95% CI	*P*
PDE4D7 score	0.37	0.23–0.58	<0.0001
ISUP pGleason Grade Group	1.8	1.4–2.4	<0.0001
pT_stage = “pT3a”	0.45	0.14–1.4	0.19
pT_stage = “pT3b”	0.1	0.01–0.9	0.04
pT_stage = “pT3c”	0.01	0–0.28	0.006
pT_stage = “pT4”	0.36	0.04– 3.3	0.36
Extra-prostatic extension	1.6	0.3–5.9	0.63
Seminal vesicle invasion	17.1	1.4–205	0.02
Surgical margin status	0.45	0.23–0.93	0.03
Lymph node invasion	2.2	1.0–4.8	0.04

*EPE* extra-prostatic extension, *CI* confidence interval, *SVI* seminal vesicle invasion, *SMS* surgical margin status, *LNI* lymph node invasion, *HR* hazard ratio.

The tested endpoint was time to prostate cancer-specific mortality (*N* = 42 events). The PDE4D7 score and the ISUP Gleason grade group were used as continuous variables; the pathology-stage pT was used as a categorical variable with pT2 as a reference. EPE, SVI, SMS and LNI were used as binary variables (0=’no’; 1=’yes’). The HR, 95% CI of the HR, and *P* values are indicated.

**Fig. 1:**
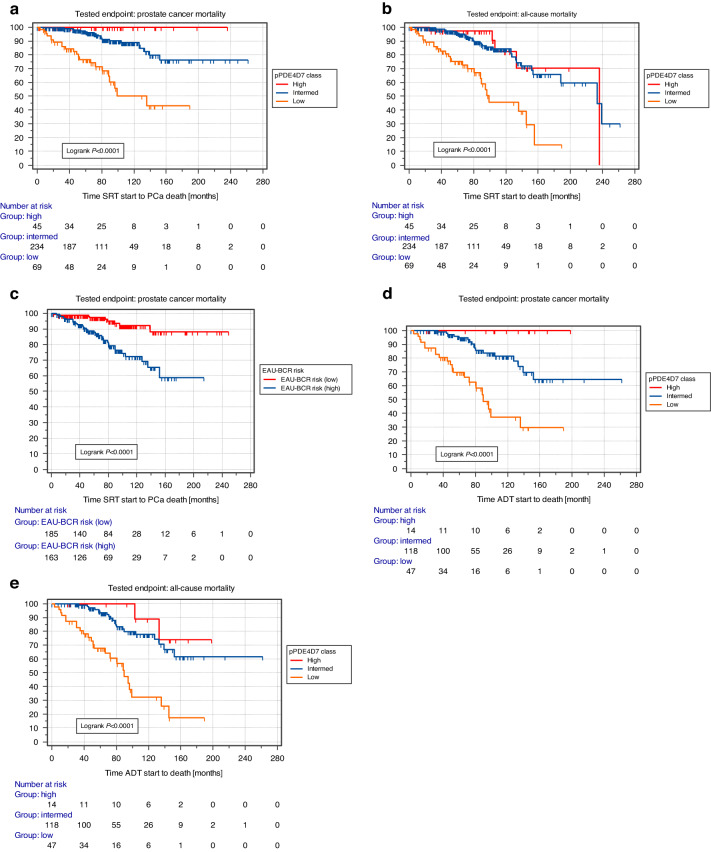
Survival analysis of the PDE4D7 score and prostate cancer-specific mortality (PCSM) after the start of salvage radiation therapy (SRT). Two cut-offs for the pPDE4D7 score were defined by AUROC (area under the ROC curve) analysis with 5-year prostate cancer-specific death after the start of salvage radiation therapy (SRT) as the dependent variable and the pPDE4D7 score as the independent variable. One cut-off (pPDE4D7 > 0.2) was defined as the point in the AUROC with maximum sensitivity and specificity. The cut-off was defined as max sensitivity (pPDE4D7 > 0.87). Consequently, the pPDE4D7 score classes stratified patients into three sub-cohorts (pPDE4D7 scores >0.87: ‘high’; pPDE4D7 scores >0.2 and <=0.87: ‘intermediate’, and pPDE4D7 scores <=0.2: ‘low’). **a** Kaplan–Meier survival analysis of the time to prostate cancer-specific mortality (PCSM) after the start of SRT in the *N* = 348 clinical high-risk prostate cancer patient cohort. Patient groups are stratified according to their PDE4D7 score, with high scores being associated with a lower risk of death due to prostate cancer than low PDE4D7 scores. **b** Kaplan–Meier survival analysis of the time to all-cause mortality (ACM) after the start of SRT in the *N* = 348 clinical high-risk prostate cancer patient cohort. **c** Kaplan–Meier survival analysis of the time to PCSM after the start of SRT in the *N* = 348 clinical high-risk prostate cancer patient cohort. Patient groups are stratified according to the EAU-BCR Risk Score, which stratifies patients into a low- vs. a high-risk class. **d** Kaplan–Meier survival analysis post-SRT of the time to PCSM after the start of ADT in a *N* = 179 prostate cancer patient cohort. **e** Kaplan–Meier survival analysis post-SRT of the time to ACM after the start of ADT in a *N* = 179 prostate cancer patient cohort. Log-rank *P* values are indicated.

Kaplan–Meier (KM) analysis of pPDE4D7 percentile scores stratified patients into three sub-cohorts with significantly different survival rates post-SRT (HR intermediate–high: NA; HR low–intermediate: 4.9; 95% CI 2.1–11.3; log-rank *P* < 0.0001; Fig. 1a). Patients with high pPDE4D7 score had a 100% 10-year survival rate, while those with intermediate and low scores had 10-year survival rates of ~90% and <50%, respectively (Fig. 1a). ACM exhibited similar trends in this patient cohort (Fig. 1b). The EAU-BCR stratified low-risk group had a significantly better survival rate at 10 years post-SRT (greater than 90%) compared to the high-risk group (~72%) (HR = 3.7; 95% CI 2.0–6.8; log-rank *P* < 0.0001; Fig. 1c).

We compared the PDE4D7 score to individual clinical parameters and clinical models, as well as combination models incorporating the PDE4D7 score with the EAU-BCR risk score and additional clinical variables, to assess the predictive ability of the PDE4D7 score for 5-year PCSM after SRT. The individual clinical parameters and models demonstrated AUCs below 0.7, while the PDE4D7 & clinical combination models exhibited AUCs of 0.81 (PDE4D7_EAU-BCR) and 0.88 (full risk model) (*P* < 0.0001). These results suggest that the PDE4D7 & clinical combination models have superior predictive ability for 5-year PCSM (Supplementary Table 1D).

Finally, KM survival analysis revealed a statistically significant difference in survival outcomes between pPDE4D7 score classes (HR intermediate–high: NA; HR low–intermediate: 4.3; 95% CI 1.9–9.6; log-rank *P* < 0.0001; Fig. 1d) in patients receiving both ADT and SRT. Specifically, patients with low pPDE4D7 score had a median survival of 89.6 months and an overall cancer-specific survival rate of 30%, while those with intermediate and high pPDE4D7 scores had not reached median survival. A similar pattern was observed for ACM (Fig. 1e). In contrast, none of the other studied risk classification models were able to significantly stratify patients (Supplementary Fig. 2A–C).

Our data demonstrate that the PDE4D7 score is a significant predictor of PCSM and ACM in patients with recurrent and progressive PCa following primary surgical resection. Patients with higher PDE4D7 scores had a more favourable long-term survival rate, while those with lower scores exhibited a poorer survival prognosis following postsurgical treatments. These findings suggest that the PDE4D7 score may be a useful predictor of outcomes in this patient population.

### PDE4D7 knockdown is associated with loss of AR signalling, enrichment of EMT and NED

**Fig. 2:**
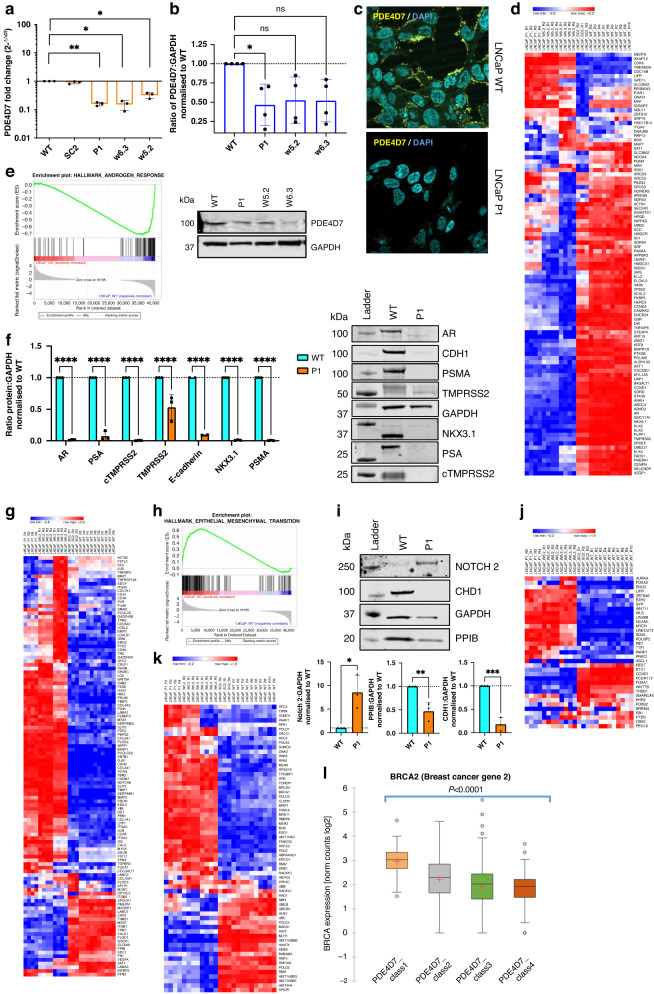
Generation of PDE4D7-knockdown LNCaP cell lines and differences in PCa-related genes. **a** PDE4D7 mRNA expression via qRT-PCR. Fold change relative to LNCaP WT (2^-∆∆Ct^) for shRNA-scrambled (SC2) and shRNA-PDE4D7 (P1, w5.2 and w6.3). **b** PDE4D7 protein expression in LNCaP WT and shRNA-PDE4D7 (P1, w5.2 and w6.3). **c** Immunocytochemistry staining of PDE4D7 protein expression (yellow) in WT and P1 LNCaPs. **d** Heatmap of gene set enrichment analysis (GSEA) hallmark androgen response in LNCaP knockdown cell lines (P1, w5.2, w6.3) vs LNCaP WT and scrambled shRNA control (SC2) based on RNAseq expression data. For all heatmaps, each RNAseq experiment was performed in multiple replicates as indicated (Rx). Gene expression TPM values were transformed to z-scores before calculating the heatmap (https://software.broadinstitute.org/morpheus). Hierarchical clustering was used to order the analysed genes and cell line samples of the gene expression matrix (metric: Euclidean distance; linkage method: average). **e** Enrichment plot GSEA hallmark androgen response in LNCaP knockdown (‘LNCaP_KD’) cell lines (P1, w5.2, w6.3) vs control LNCaP cell lines (WT and SC2; together ‘LNCaP_WT’). The enrichment score (ES) is indicated by the green line demonstrating enrichment of expression of androgen response genes in the LNCaP WT cell lines vs. a depletion of expression in the three PDE4D7-knockdown cell lines clones P1, w5.2, w6.3; NES (Normalised Enrichment Score)=-2.1; FDR *q* value < 0.001. **f** Western blot and quantification of the protein expression of prostate cancer-related RNAseq identified genes in WT and P1 LNCaPs. **g** Heatmap of GSEA hallmark epithelial–mesenchymal transition (EMT) in LNCaP knockdown cell lines (P1, w5.2, w6.3) vs LNCaP WT and SC2. **h** Enrichment plot GSEA hallmark epithelial–mesenchymal transition (EMT) in LNCaP knockdown (‘LNCaP_KD’) cell lines (P1, w5.2, w6.3) vs LNCaP control cell lines (WT and SC2; together ‘LNCaP_WT’). The enrichment score (ES) is indicated by the green line demonstrating enrichment of expression of EMT genes in the three PDE4D7-knockdown cell line clones P1, w5.2, and w6.3 vs. LNCaP WT and LNCaP SC2; NES (Normalised Enrichment Score)=1.9; FDR *q* value < 0.001. **i** Western blot and quantification of the protein expression of EMT-related RNAseq identified genes in WT and P1 LNCaPs. **j** Heatmap of neuroendocrine differentiation (NED) involved genes in LNCaP knockdown cell lines (P1, w5.2, w6.3) vs LNCaP WT and SC2. **k** Heatmap of genes involved in homology-directed DNA repair pathways (Supplementary Table 5) in LNCaP knockdown cell lines (P1, w5.2, w6.3) vs LNCaP wild-type (WT) and scrambled shRNA control (SC2). **l** Expression boxplot of the BRCA2 gene in PDE4D7 expression score classes in human clinical patient samples. The expression of each gene is provided after TPM calculation based on the RNAseq count data. The PDE4D7 classes represent different categories of PDE4D7 expression based on the PDE4D7 score (see references), where PDE4D7_class1 (most left-hand box) represents the lowest PDE4D7 scores (i.e., lowest PDE4D7 expression), while PDE4D7_class4 (most right-hand box) represents the highest PDE4D7 scores (i.e., highest PDE4D7 expression). The number of patients per class are as follows: PDE4D7_class1 (*N* = 13); PDE4D7_class2 (*N* = 134); PDE4D7_class3 (*N* = 301); PDE4D7_class4 (*N* = 85). The median of the expression per group is indicated by the bar within each box. The red cross represents the mean expression value per group. The circles represent outlier expression values. The *P* values were calculated by use of ANOVA Kruskal–Wallis test and represent a significant change in expression over the four PDE4D7 classes.

To further investigate the biological significance of PDE4D7 in PCa development and progression, we generated a derivative of the LNCaP (clone FCG) cell line with selective knockdown of PDE4D7 expression using lentivirus-mediated shRNA (Supplementary Fig. 3A). Among several PDE4D-knockdown LNCaP clones, LNCaP_P1 cells exhibited the most significant downregulation of PDE4D7 mRNA expression, with no decrease observed in scrambled shRNA control (LNCaP_SC2) cells (Fig. 2a). Western blot analysis and confocal microscopy further confirmed the reduction in PDE4D7 protein expression in LNCaP_P1, LNCaP_w5.2 and LNCaP_w6.3 cells compared to wild type (LNCaP_WT) (Fig. 2b, c and Supplementary Fig. 4C). Importantly, neither PDE4D5 nor PDE4D9 were inadvertently knocked down in the shRNA-PDE4D7 clones (Supplementary Fig. 4A, B). These findings demonstrate the successful generation of a selective PDE4D7-knockdown model in LNCaP cells.

We performed next-generation sequencing (NGS) RNA sequencing of the two control cell lines (LNCaP_WT and LNCaP_SC2) as well as three PDE4D7-knockdown clones (LNCaP_P1, LNCaP_w5.2 and LNCaP_w6.3) and analysed the 50 GSEA hallmark pathways (gsea-msigdb.org). We identified the AR response pathway as the most significantly depleted gene set in the PDE4D7-knockdown cell lines (false discovery rate (FDR) *q* value < 0.001; normalised enrichment score −2.1; Fig. 2d, e and Supplementary Table 2). To confirm that genomic changes were reflected at the protein level, western blotting for selected PCa-related proteins was performed, with significant downregulation observed in the P1 cell line compared to LNCaP WT (Fig. 2f). Importantly, the scrambled shRNA cell line (SC2) did not show notable differences in the expression of these genes (Supplementary Fig. 5). Among the most downregulated genes in the three PDE4D7-knockdown cell lines were the known AR-regulated kallikrein-related peptidases KLK2 and KLK3, transmembrane serine protease 2 (TMPRSS2), and the transcription factor NK3 homeobox-1 (NKX3–1). In addition, the AR itself was significantly downregulated in LNCaP P1 cells (Fig. 2f). Loss of GRK2 has been associated with non-AR-driven PCa expression. Whilst the expression of this GPCR was significantly downregulated on the transcript level between the WT and knockdown cell lines, the expression seemingly remained unchanged on the protein level (Supplementary Fig. 12). The described findings indicate that the selective depletion of PDE4D7 leads to an androgen-insensitive cellular phenotype reflecting progressive PCa.

In contrast, we identified multiple hallmark pathways significantly enriched in the PDE4D7-knockdown LNCaPs. Of those, the EMT pathway demonstrated the highest level of enrichment (FDR *q* value < 0.001; normalised enrichment score 1.9; Fig. 2g–i and Supplementary Table 3). Other clinically relevant hallmark pathways with FDR *q* values < 0.1 are WNT beta-catenin and hedgehog signalling, both of which have been linked to PCa progression after the development of hormone resistance [27, 28].

Neuroendocrine differentiation (NED) is a hallmark of aggressive, AR-independent and treatment-resistant PCa, and can occur in both primary and metastatic disease [29, 30]. NED is characterised by decreased AR expression and increased expression of neuroendocrine markers [31]. Analysis of our LNCaP models revealed that many genes with previously reported induced expression levels in neuroendocrine prostate cancer (NEPC) were upregulated in the PDE4D7-knockdown LNCaPs (Supplementary Table 4), including classical NED markers such as ENO2, SYP, AURKA and NCAM1 (Fig. 2j).

To explore the potential clinical relevance of these findings, we also analysed the expression of hallmark AR response genes and key markers for NED in RNAseq data of 533 human patient samples described previously [32] and found that all tested AR response genes were downregulated alongside reduced PDE4D7 expression in these patient tumours, while the expression of AR itself did not change (Supplementary Fig. 6A). Similarly, we found altered expression of several NED markers between low vs high PDE4D7 tumours (Supplementary Fig. 6B), consistent with previously reported alterations in NEPC [29, 33].

### PDE4D7 knockdown reflects aggressive phenotype and confers resistance to AR inhibition

**Fig. 3:**
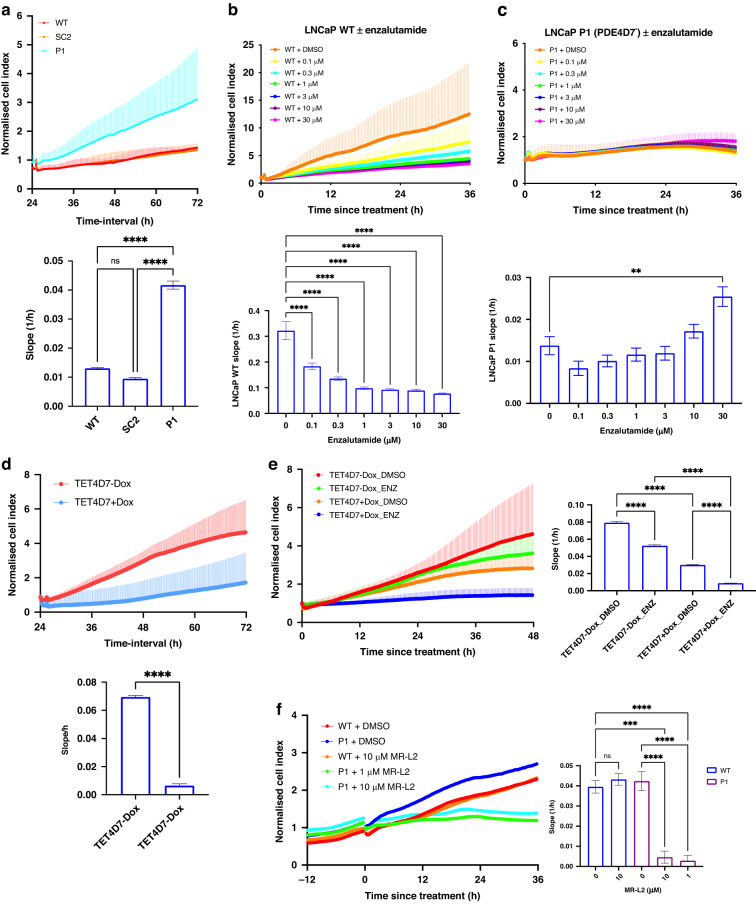
PDE4D7 knockdown enhances growth and confers enzalutamide resistance, which is rescued upon PDE4D7 re-expression. **a** Real-time proliferation of LNCaP WT, shRNA-scrambled (SC2) and shRNA-PDE4D7 (P1) cells. **b**, **c** Real-time analysis of LNCaP WT (**b**) and LNCaP P1 (**c**) cells treated with enzalutamide (0 µM–30 µM) or 0.1% DMSO. **d** Real-time growth analysis of P1-TET4D7 +/− doxycycline induction of PDE4D7 alone or **e** in response to enzalutamide 10 µM. **f** Real-time growth analysis of WT and P1 LNCaP cells upon treatment with the PDE4 activator MR-L2 (1–10 µM). Slope analysis from 0 to 48 h post treatment. Mean +/− SEM, *N* = 3, ***P < 0.001, ****P < 0.0001, ns non-significant.

Knockdown of PDE4D7 resulted in increased growth characteristics in comparison to WT and SC2 LNCaP cells (Fig. 3a). Importantly, no significant difference in growth was found between the WT and SC2 cells. In addition, we have been able to correlate level of PDE4D7 knockdown to proliferative capacity of LNCaP cells, whereby the w5.2 clone showed slightly reduced growth to P1 (Supplementary Fig. 4D) whilst the w6.3 clone revealed slightly enhanced growth (Supplementary Fig. 4E), correlating with slightly enhanced PDE4D7 knockdown in w6.3, and weakest level of PDE4D7 knockdown in w5.2, in comparison to the P1 LNCaP clone (Fig. 2a, b). These data may be consistent with the concept of clonal differences represented by clinically relevant tumour heterogeneity.

Enzalutamide, an AR inhibitor used to treat advanced PCa [34], was utilised to investigate the effects on the growth of PDE4D7-knockdown LNCaPs. Dose-response experiments showed a significant decrease in WT proliferation at all concentrations tested (0.1–30 µM) (Fig. 3b). In contrast, P1 LNCaPs showed minimal reduction in proliferation and even exhibited enhanced growth at higher doses (Fig. 3c). Induced re-expression of PDE4D7 in P1 LNCaPs (Supplementary Fig. 3B) rescued the growth phenotype and restored sensitivity to enzalutamide (Fig. 3d, e). These findings strongly highlight that low PDE4D7 expression in PCa cells confers resistance to anti-androgens such as enzalutamide.

MR-L2 is a PDE4 longform activator that binds allosterically to the PDE4 enzyme to mimic PKA phosphorylation in the UCR1 domain [35]. Activation of PDE4 in P1 LNCaPs rescued the enhanced growth phenotype caused by PDE4D7 knockdown, whilst having no effect on the proliferation of WT LNCaPs (Fig. 3f). These data again highlight the importance of PDE4D7 in driving PCa proliferation.

### PDE4D7 knockdown is associated with alterations in DNA damage repair genes

Genomic alterations in DNA damage repair (DDR) genes are prevalent in both primary and metastatic PCa tissue [36], leading to the clinical utilisation of PARP inhibitors (PARPi) in patients with DDR deficiencies, however, the emergence of PARPi resistance has been observed [37, 38]. To investigate the potential impact of PDE4D7 knockdown on DNA repair mechanisms, we used the REACTOME homology-directed repair (HDR) of the DNA double-strand breaks gene set (reactome.org: R-HSA-5693538) and expanded it with additional genes from other DDR pathways previously reported to be altered in PCa (Supplementary Table 5) [36]. Our analysis revealed alterations in the expression of a subset of DDR genes in PDE4D7-knockdown cells, particularly those commonly mutated in PCa. Many of these genes were significantly upregulated in PDE4D7-knockdown cells (Fig. 2k), while we did not identify significant differences in single nucleotide variants between the parent and knockdown cell lines (Supplementary Fig. 7A, B, respectively).

We then reanalysed RNAseq data from 533 clinical samples [32] focusing on the expression of PCa-relevant DDR genes, revealing the most significant increase in BRCA2 expression with decreasing PDE4D7 expression (*P* < 0.0001), along with a range of other DDR-related genes (Fig. 2l and Supplementary Fig. 8). BRCA1, ATM and BRIP1 were also upregulated, although non-significantly. Based on these findings, we posit that the altered expression of DDR genes with downregulated PDE4D7 may contribute resistance to DDR-targeting therapies, therefore PDE4D7-knockdown LNCaPs may exhibit resistance to PARPi compounds such as olaparib.

### PDE4D7 re-expression in PDE4D7-knockdown LNCaPs leads to molecular changes reflecting a less aggressive phenotype

**Fig. 4:**
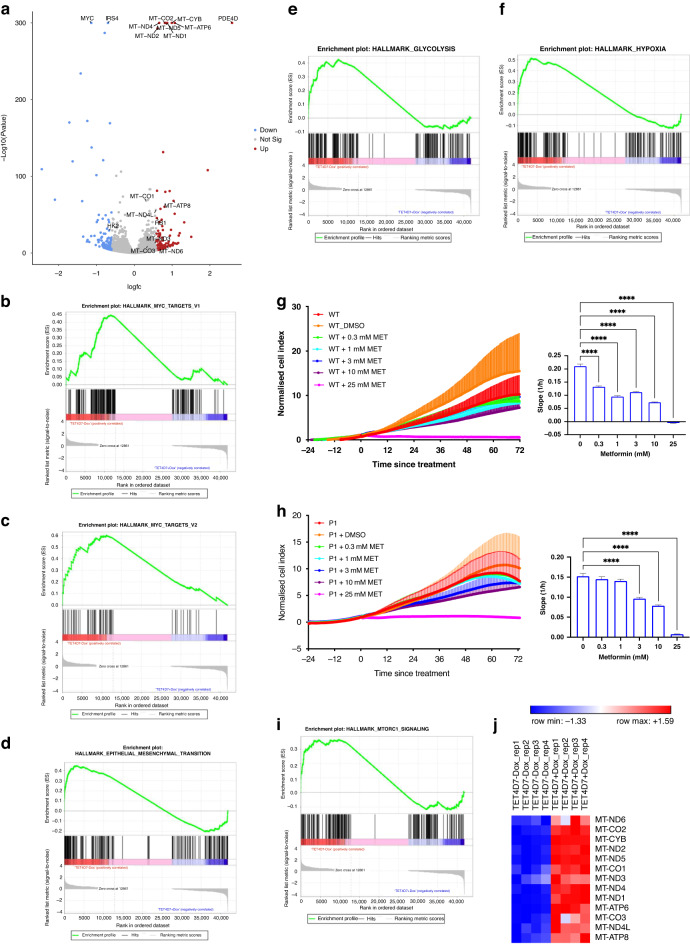
Re-expression of PDE4D7 rescues many hallmark pathways previously enriched in PDE4D7-knockdown LNCaPs. **a** Volcano plot of differentially expressed genes (DEGs) in LNCaP P1, PDE4D7 Tet inducible cell lines; ‘LNCaP_P14D7Tet_min’ (without doxycycline stimulation) vs ‘LNCaP_P14D7Tet_plus’ (with doxycycline stimulation). DEGs are shown in red (up in ‘LNCaP_P14D7Tet_plus’) or blue (down in ‘LNCaP_P14D7Tet_plus’) if the log2-fold change (logFC) change in expression was >1.5 or <−1.5 and the the adjusted *P* value > 0.05. All other genes are represented as grey dots. The top 25 significant DEGs are annotated with their gene symbols. Note: the plot only includes protein-coding genes. **b**, **c** Enrichment plot GSEA hallmark MYC targets V1 (**b**) and V2 (**c**) in ‘LNCaP_P14D7Tet_min’ vs ‘LNCaP_P14D7Tet_plus’. The enrichment scores (ES) are indicated by the green line demonstrating enrichment of expression of MYC target genes in the ‘LNCaP_P14D7Tet_min’ vs ‘LNCaP_P14D7Tet_plus’ cell line; NES (Normalised Enrichment Score)=1.5; FDR *q* value = 0.03, and 1.5; FDR *q* value = 0.003 for hallmark MYC targets V1, and V2, respectively. **d** Enrichment plot GSEA hallmark epithelial–mesenchymal transition (EMT). The enrichment score (ES) demonstrates an enrichment of expression of EMT genes in the ‘LNCaP_P14D7Tet_min’ vs ‘LNCaP_P14D7Tet_plus’ cell line; NES (Normalised Enrichment Score)=1.5; FDR *q* value = 0.03. **e** Enrichment plot GSEA hallmark Glycolysis. The enrichment score (ES) demonstrates an enrichment of expression of glycolysis involved genes in the ‘LNCaP_P14D7Tet_min’ vs ‘LNCaP_P14D7Tet_plus’ cell line; NES (Normalised Enrichment Score)=1.4; FDR *q* value = 0.04. **f** Enrichment plot GSEA hallmark Hypoxia. The enrichment score (ES) demonstrates an enrichment of expression of hypoxia-involved genes in the ‘LNCaP_P14D7Tet_min’ vs ‘LNCaP_P14D7Tet_plus’ cell line; NES (Normalised Enrichment Score)=1.75; FDR *q* value = 0.004. **g**, **h** Real-time growth analysis of WT (**g**) and PDE4D7-knockdown P1 (H) LNCaPs upon treatment with 0.3–25 mM metformin or 0.1% DMSO. Normalised cell index to treatment timepoint. Slope analysis 0–48 h post treatment. Statistical analysis via one-way ANOVA (mean +/− SEM, *N* = 3, ****P < 0.0001). **i** Enrichment plot GSEA hallmark MTORC1 signalling. The enrichment score (ES) demonstrates an enrichment of expression of mTORC signalling involved genes in the ‘LNCaP_P14D7Tet_min’ vs ‘LNCaP_P14D7Tet_plus’ cell line; NES (Normalised Enrichment Score)=1.24; FDR *q* value = 0.179. **j** Heatmap of mitochondrial protein-coding genes in PDE4D7 inducible LNCaP P1 knockdown cell line TET4D7 (TET4D7-Dox: -doxycycline; TET4D7+Dox: +doxycycline). For all heatmaps, each RNAseq experiment was performed in multiple replicates. Gene expression TPM values were transformed to z-scores before calculating the heatmap (https://software.broadinstitute.org/morpheus). Hierarchical clustering was used to order the analysed genes and cell line samples of the gene expression matrix (metric: Euclidean distance; linkage method: average).

RNA sequencing of PDE4D7 re-expressing LNCaP P1 cells led to significant downregulation of MYC (Fig. 4a), a frequently overexpressed gene and a critical driver of progression in PCa [39]. This downregulation was confirmed by GSEA using MYC targets hallmark gene sets (Fig. 4b, c and Supplementary Table 6). MYC overexpression has been shown to reduce the transcriptional activities of the AR and may cause treatment resistance and disease progression [39]. The significant downregulation of MYC in LNCaP P1 upon PDE4D7 re-expression (logFC=0.45; *P*≪0.0001) may reprogramme the cell line to AR transcription and subsequently re-sensitise to AR inhibitors.

In addition, the EMT hallmark gene set, which was highly enriched in PDE4D7-knockdown LNCaPs compared to WT, was significantly depleted after PDE4D7 re-expression (Fig. 4d). This finding suggests that PDE4D7 plays a crucial role in regulating the EMT pathway, a hallmark of cancer progression and metastasis.

Interestingly, PDE4D7 re-expression also significantly upregulated several mitochondrial genes (Fig. 4a). This finding is noteworthy because progressively growing cancer cells typically rely on glycolysis leading to decreased ATP generation [40, 41]. This is supported by the significant depletion of the oxidative phosphorylation hallmark gene set after PDE4D7 knockdown (Supplementary Table 2) while the hypoxia hallmark gene set is enriched (Supplementary Table 3) which has been reported to increase glycolysis [42]. In contrast, upon PDE4D7 re-expression, P1 LNCaPs significantly downregulated HK2 (logFC=0.63; *P*«0.0001; Fig. 4a) and depleted the glycolysis and hypoxia hallmark gene sets (Fig. 4e, f). HK2 is a key glycolytic enzyme and a key regulator of switching the energy metabolism of tumour cells from glycolysis back to mitochondrial respiration [43]. In addition, all 13 protein-coding mitochondrial genes which all encode protein subunits of the enzyme complexes of the oxidative phosphorylation system are strongly upregulated upon PDE4D7 doxycycline-driven induction (Fig. 4a, j), suggesting that PDE4D7 re-expression promotes switching from glycolysis to oxidative phosphorylation as the main metabolic pathway to process glucose.

Metformin is a type II diabetes drug known to inhibit complex I of the mitochondrial respiratory oxidative phosphorylation chain [44]. Interestingly, metformin had a significant anti-proliferative effect on WT LNCaPs, but not on P1 LNCaPs unless treated with supra-physiological concentrations (Fig. 4g, h), which may be due to the enhanced oxidative phosphorylation in WT cells. Additionally, complex I inhibition leads to AMPK activation and inhibition of the mTOR pathway which is known to play a key role in tumour development and progression [44, 45]. The hallmark gene set MTORC1 was significantly depleted in LNCaP P1 cells (FDR *q* value = 0.006; normalised enrichment score = −1.6; Fig. 4i), which may indicate that the susceptibility of WT LNCaPs to metformin is based on inhibition of mTOR signalling. Downregulated mTOR signalling upon PDE4D7 knockdown may subsequently lead to metformin resistance. It is of note though that PDE4D7 re-expression does not lead to re-activation of the MTORC1 pathway in LNCaP P1 cells (Supplementary Table 6).

Taken together, this data provides important insights into the regulatory role of PDE4D7 in MYC regulation, energy metabolism, and EMT, culminating in a less aggressive phenotype.

### PDE4D7 knockdown confers resistance to PARP inhibition and PDE4D7 re-expression enhances sensitivity to olaparib and docetaxel

**Fig. 5:**
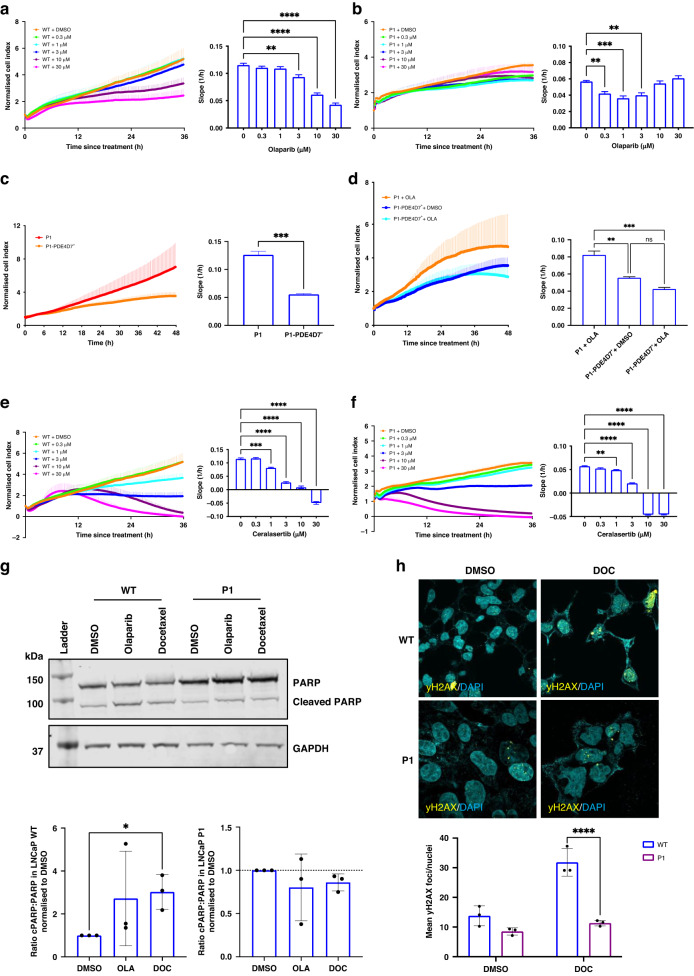
PDE4D7 knockdown in LNCaP cells enhances resistance to olaparib. **a**, **b** Real-time proliferation of LNCaP WT (**a**) and shRNA-PDE4D7 (P1) cells following treatment with olaparib (0 µM–30 µM) or 0.1% DMSO. Normalised cell index to timepoint of treatment. **c**, **d** Real-time proliferation of P1 and LNCaP P1-PDE4D7^+^ cells in response alone (**c**) or in response to 10 µM olaparib (**d**). **e**, **f** Real-time proliferation of LNCaP WT (**e**) and P1 (**f**) cells with ceralasertib (0 µM–30 µM). Slope analysis 0–30 h post treatment. Normalised cell index to timepoint of treatment. Mean ± SEM, *N* = 3, *P < 0.05, **P < 0.01, ***P < 0.001, ****P < 0.0001. **g** Western blot analysis of PARP and cleaved PARP (cPARP) in WT and P1 LNCaPs following 48 h treatment with 10 µM Olaparib or 10 nM docetaxel. **h** Immunostaining of γ-H2AX foci in WT and P1 LNCaPs following treatment with 10 nM docetaxel of 0.1% DMSO.

Given the alterations in DDR genes between WT and P1 LNCaPs, these cells were tested in response to olaparib (PARPi) and ceralasertib (ATR inhibitor), both of which have been shown effective as PCa treatments [46, 47]. LNCaP WT proliferation showed significant reductions at 3, 10 and 30 µM olaparib (Fig. 5a), whereas the effect on LNCaP P1 was minimal yet with unexpected reduction at low concentrations (Fig. 5b). It is notable however, that cells with reduced PDE4D7 expression were resistant to olaparib at 10 and 30 µM (Fig. 5b). Furthermore, as with the TET-On induction of PDE4D7, transiently transfected PDE4D7 into P1 LNCaPs (P1-PDE4D7^+^) significantly downregulated growth (Fig. 5c) and re-sensitised cells to 10 µM olaparib (Fig. 5d). These data reconfirm the role of PDE4D7 in blunting PCa cell growth and promoting susceptibility to clinically relevant therapeutics.

While PDE4D7 knockdown conferred resistance to 10–30 µM olaparib, these cells were extremely sensitive to ceralasertib at these concentrations with similar responses between WT and P1 LNCaPs (Fig. 5e, f). Similarly, docetaxel significantly reduced growth of both WT and P1 LNCaPs (Supplementary Fig. 9A, B, respectively), however, P1 LNCaPs began to regain their proliferative potential ~36 h post treatment, particularly with higher docetaxel concentrations, suggesting that these cells may be able to ameliorate docetaxel-mediated DNA damage and regrow. Consistent with these findings, analysis of the cleaved PARP:PARP ratio revealed induction of apoptosis in WT LNCaPs in response to both olaparib and docetaxel, whereas no influence on apoptosis was observed in P1 LNCaPs (Fig. 5g). Given that cells were treated for 48 h prior to analysis of cPARP:PARP, and that P1 LNCaP growth began to steadily rise, it appears that the initial docetaxel-mediated reduction in growth of these cells did not lead to induction of late stage apoptosis-like in the WT LNCaPs, suggesting a level of resistance to docetaxel upon PDE4D7 knockdown in LNCaPs.

Furthermore, as with olaparib, reintroduction of PDE4D7 into P1 LNCaPs further enhanced sensitivity to docetaxel (Supplementary Fig. 9C). In correlation with this, γ-H2AX assay revealed an upregulation in DSBs as measured by number of γ-H2AX foci upon docetaxel treatment in WT LNCaPs compared to P1 LNCaPs (Fig. 5h).

Overall, these findings suggest that a paucity of PDE4D7 promotes the ability to repair associated DNA damage and protects against the induction of cell death.

## Discussion

### PDE4D7 score predicts PSA relapse, mortality and therapy response

Patients with PCa rely on treatment stratification to receive the most efficacious therapy for their specific disease presentation. SRT remains a commonly utilised treatment modality for PSA recurrent disease, however, reported 5-year biochemical failure rates range between 25 and 70% [48]. Further disease progression after SRT failure is driven by AR signalling [49], hence ADT has remained a mainstay treatment for advanced disease for several decades [50]. While initially responsive to ADT, it may select for cellular clones that evade the selection pressure via androgen-independent survival mechanisms, inevitably leading to the development of ADT-resistant CRPC [51]. Therefore, it is imperative to identify early emerging treatment resistance to select alternative treatment regimens and improve survival outcomes. This may include combination therapies such as ADT in conjunction with second-generation anti-androgens or docetaxel [52–54].

Prognostic genomic biomarkers that objectively assess the risk of death from advanced PCa may aid in the selection of patients for treatment de-escalation (those at virtually no risk of PCSM) versus treatment intensification (those with poor prognosis). Clinical trials are currently evaluating molecular stratification and prognostic tools in determining the optimal treatment approach for high-risk/recurrent PCa patients [55–57], while the PREDICT-RT trial (NCT04513717) examines the feasibility of less intense treatment for genomic low-risk patients.

This study demonstrates that individuals with low PDE4D7 expression exhibit a substantially elevated risk of PCSM. Alterations in PDE expression result in dysregulated cAMP/PKA signalling which has been extensively linked to PCa and therapy resistance. PDE4 is one of the most highly expressed PDE families in the human prostate [58], consistent with our previous studies [20]. Baca et al. proposed that PCa progresses through clonal and punctuated events rather than gradual or catastrophic evolution, and identified the loss of PDE4D as an early, clonal event in cancer development [59]. In addition, a study that employed whole-genome sequencing of 112 primary and metastatic PCa samples identified PDE4D as a potential driver gene of the disease [60]. The PDE4D gene was often impacted by chromosomal rearrangements and regions of loss of heterozygosity, particularly in the part encoding the long PDE4D transcripts such as PDE4D7. The study demonstrated that homozygous deletions on chromosome 5 occur more frequently and earlier in the disease course in ETS fusion-negative tumours compared to ETS fusion-positive. In addition, our previous study reported that TMPRSS2-ERG fusion impacts progression-free survival as determined by PDE4D7 expression [32]. Intriguingly, our data here show that low expression of PDE4D7 is associated with a strongly elevated risk of death in ETS fusion-negative tumours (Supplementary Fig. 10A, B). Taken together, these findings suggest that loss of PDE4D7 may represent an early, clonal event in ETS fusion-negative PCa and provides significant evidence that PDE4D7 expression is involved in PCa progression and is inversely associated with progression-free and disease-specific survival after prostate-specific antigen relapse.

Therapy resistance in PCa may be attributed to the intrinsic biology of the tumour or may develop upon therapeutic interventions. One potential mechanism of resistance is the development of a neuroendocrine phenotype via NED in primary adenocarcinoma, which is the most common histopathology of primary PCa. This phenomenon has been observed to occur both in treatment-naive tumours and in tumours that have undergone ADT [61]. In untreated tumours, cells that stain positively for neuroendocrine markers are referred to as NE-like [62]. However, it is well established that PCa cells in culture can undergo NED in vitro through exposure to various stimuli, such as increases in cAMP and/or PKA activation [63]. In treated tumours, it is believed that NED is driven by selection pressure induced by treatment [64, 65] associated with reduced AR activity or expression [66]. It has been postulated that differentiated NE-like cells may progress to small-cell prostate carcinoma (SCPC) through the accumulation of additional genetic alterations, such as loss of RB1, MYCN, and AURKA amplification [61]. These cells are characterised by rapid proliferation, loss of AR and PSA expression, enhanced transcription of neuroendocrine markers, and further therapeutic resistance [29].

**Fig. 6:**
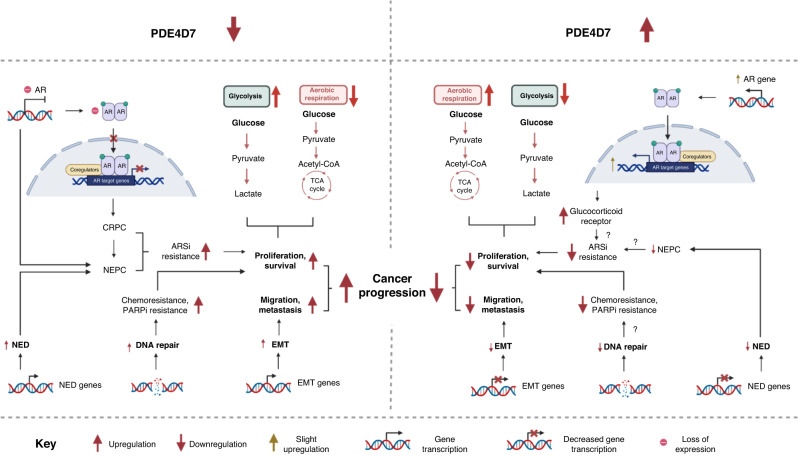
PDE4D7 loss leads to many biological changes promoting cancer progression, of which can be reversed with re-expression of PDE4D7. Schematic diagram showing the genetic and biological pathway changes upon PDE4D7 downregulation (left) and PDE4D7 re-expression in PDE4D7-low (right) PCa cells. (Created with BioRender.com). Note that the re-expression of the AR and AR response genes after PDE4D re-expression was small compared to the original levels of the expression of these genes in the wild-type cell line and were mostly seen in the cell line with stable PDE4D7 re-expression (i.e., less pronounced in the inducible cell line).

Our findings demonstrate that targeted disruption of the PDE4D7 transcript results in the emergence of a phenotype that mimics SCPC. Notably, AR and its associated response genes are downregulated, while cells exhibit accelerated proliferation and resistance to a broad range of therapeutic compounds, as schematically outlined in Fig. 6. These transcriptomic alterations are partially replicated in patient samples with low PDE4D7 expression. We postulate that the reduction in PDE4D7 transcription results in a pre-differentiation state to NEPC, which can subsequently progress to SCPC under conditions of hormone deprivation. This could contribute to treatment resistance and poor prognosis, as demonstrated in patients with diminished PDE4D7 expression in their primary tumours. Based on these findings, we propose that PDE4D7 may serve as a biomarker for the early identification of NEPC. In addition, we suggest close monitoring of patients with low PDE4D7 expression who are treated with potent anti-androgens, as these tumours may rapidly progress to a state where further treatment options become limited.

### PDE4D7 expression influences the DNA damage response

An important aspect of treatment resistance that we investigated is the alteration of DDR pathways. Inhibition of PARP1/2 activity has become a cornerstone of treatment for advanced cancers with DDR deficiencies and has been extensively studied in the literature [67]. However, it has also been reported that an increase in the gene expression level of DDR genes may lead to resistance to cisplatin chemotherapy [68], while the overexpression of BRCA1, BRCA2, RAD51 and RPA1 has been observed in hypopharyngeal and nasopharyngeal carcinoma cells resistant to radiotherapy [69].

We present evidence that the selective suppression of PDE4D7 in LNCaP cells results in the transcriptional induction of various DDR-related genes, which is replicated to some extent in patient samples with low PDE4D7 expression. This may explain the resistance of PDE4D7-knockdown LNCaPs to the olaparib and decreased sensitivity to docetaxel. Interestingly, the PARPi-resistant PDE4D7 knockdown cells were substantially susceptible to ATR inhibition, which could provide a potential future therapeutic option for PARPi-resistant tumours.

A recent publication showed that oncogene-driven activation of cAMP signalling in pituitary somatotroph adenomas induces double-strand break DNA damage. The DNA damage may be further augmented by the reported downregulation of the PDE4D gene in these tumours [70]. Furthermore, somatotroph tumours are characterised by widespread chromosomal copy number abnormalities, which may be related to the observation of extensive copy number variations in the PDE4D7-knockdown LNCaPs in comparison to WT (Supplementary Fig. 11A–D). Interestingly, we recently reported a protein–protein interaction between PDE4D7 and the helicase DHX9. This enzyme is involved in multiple functional processes, including the resolution of R-loop DNA–RNA hybrids which are an important source of replication stress and genomic instability [71]. The downregulation of PDE4D7 may impact the capacity of DHX9 to resolve these hybrid structures which may in turn be the result of cAMP pathway-driven DNA damage in PCa cells.

### PDE4D7 re-expression impacts the cancer cell-specific metabolic reprogramming (Warburg effect)

The Warburg effect, named after Otto Warburg, refers to the observation that cancer cells often exhibit distinct metabolic behaviour compared to normal cells [40]. Specifically, cancer cells tend to rely heavily on glycolysis, a process that breaks down glucose to produce energy, even in the presence of adequate oxygen (aerobic glycolysis) [72].

The Warburg effect is characterised by increased glucose uptake and lactate production, even in the presence of sufficient oxygen. This metabolic shift allows cancer cells to meet their high energy demands and support rapid proliferation [72]. In addition to increased glucose consumption, cancer cells also show altered metabolism of other nutrients, including amino acids and lipids, to support cell growth and survival [73].

Furthermore, signalling pathways such as the MYC pathway, which are commonly dysregulated in cancer, play crucial roles in promoting the Warburg effect. These pathways stimulate glucose uptake, increase the expression of glycolytic enzymes, and enhance the synthesis of macromolecules required for cancer cell growth [74].

The Warburg effect not only provides cancer cells with energy and building blocks for growth but also contributes to other hallmarks of cancer, including immune evasion and angiogenesis. The high lactate production in cancer cells creates an acidic microenvironment that suppresses immune responses. In addition, the Warburg effect induces angiogenesis, the formation of new blood vessels, to supply nutrients and oxygen to rapidly dividing cancer cells [75].

In summary, the Warburg effect describes the metabolic reprogramming observed in cancer cells, characterised by enhanced glycolysis and lactate production, even in the presence of oxygen. This metabolic shift is driven by genetic and signalling alterations and provides cancer cells with energy and molecular building blocks for growth.

There is an ongoing debate though why cancer cells switch their use of glucose as an energy resource from oxidative phosphorylation to glycolysis under aerobic conditions despite the significant reduction of around 18x loss of ATP generation. It was recently suggested that the level of cellular uptake of glucose and the initial generation of pyruvate as the main glycolytic metabolite that enters the oxidative phosphorylation process in mitochondria would lead to a saturation of mitochondrial activity [42]. So rather than suppression of oxidative phosphorylation the process of glycolysis would become the primary source of glucose breakdown due to mitochondrial overload. The data presented here indicates that upon PDE4D7 knockdown the process of oxidative phosphorylation is diminished by depletion of the respective hallmark genes and enrichment of the hypoxia hallmark which has been reported to turn on the glycolysis pathway (Fig. 6). The re-expression of PDE4D7 reverses this effect by depletion of the glycolysis hallmark gene set (Fig. 6) and induction of expression of all mitochondrial protein-coding genes. Therefore, we hypothesize that the level of PDE4D7 expression and activity provides a switch in prostate cancer cells to reprogramming energy metabolism that today has been recognised as one of the hallmarks of cancer [75].

### PDE4D7 re-expression or activation as a therapeutic avenue

We have shown through re-expression of PDE4D7 in the knockdown LNCaP cells via either transient DNA transfection or an inducible Tet-On system that we can not only restore a reduced growth phenotype but also re-sensitise cells to treatments such as enzalutamide and olaparib, highlighting the potential for manipulating PDE4D7 gene expression as a novel therapeutic avenue for PCa, independent of AR-targeting treatments.

In addition, using a PDE4 long-form activating compound which mimics PKA phosphorylation within the UCR1 domain [35], we can reactivate the residual PDE4D7 in PCa cells and hinder proliferation. Ultimately, our findings support the feasibility of either re-activation of PDE4D7 via compounds, or enhanced expression of PDE4D7 transcripts such as via gene therapy, as a new treatment for high-risk PCa patients to reduce tumour growth and overcome therapeutic resistance in the treatment of advanced PCa.

## Conclusion

This study suggests that decreased PDE4D7 expression is associated with an aggressive PCa phenotype characterised by enhanced proliferation and therapeutic resistance. Knockdown of PDE4D7 in LNCaP cells led to reduced AR signalling, increased EMT, NED and DDR pathway alterations, partially replicating clinical findings. Tumours with low PDE4D7 expression may be at high risk for progressing towards NEPC and therapeutic resistance, suggesting caution in using potent androgen inhibitors. Targeting PDE4D7 or its downstream pathways may maintain cAMP signalling during ADT and provide a novel strategy for extending the period of androgen response in advanced PCa.

### Supplementary information

Supplementary Materials

Supplementary Table 8

Supplementary Table 9

## Data Availability

Supplementary Table 8 includes the DESeq2 results of differential gene expression for the LNCaP wild-type and SC2 cell line compared to the three PDE4D7-knockdown clones P1, w5.2 and w6.3. Supplementary Table 9 provides the DESeq2 normalised gene expression data for all cell lines/clones used in this study. The availability of the human clinical patient sample-related data is restricted by the EU and national data privacy law as well as contractual obligations. Interested parties may contact the corresponding author to discuss access options to these data.
