# Supplementary material for: Loss of PDE4D7 expression promotes androgen independence, neuroendocrine differentiation and alterations in DNA repair: implications for therapeutic strategies
Source: Br J Cancer. 2023 Sep 22;129(9):1462–76. doi: 10.1038/s41416-023-02417-5 (PMC10628190; doi:10.1038/s41416-023-02417-5)
Supplement: Supplementary file 1 — Supplementary Materials [file 41416_2023_2417_MOESM1_ESM.docx]

**Supplementary Materials**

**Supplementary Figure 1: Patient inclusion and Data Analysis Flow Overview.** Patient inclusion criteria for the study cohort (N=367 patients). The various sub-cohorts with the relevant number of patients are shown. RP – radical prostatectomy; SRT – salvage radiation therapy; ADT – androgen deprivation therapy.

**
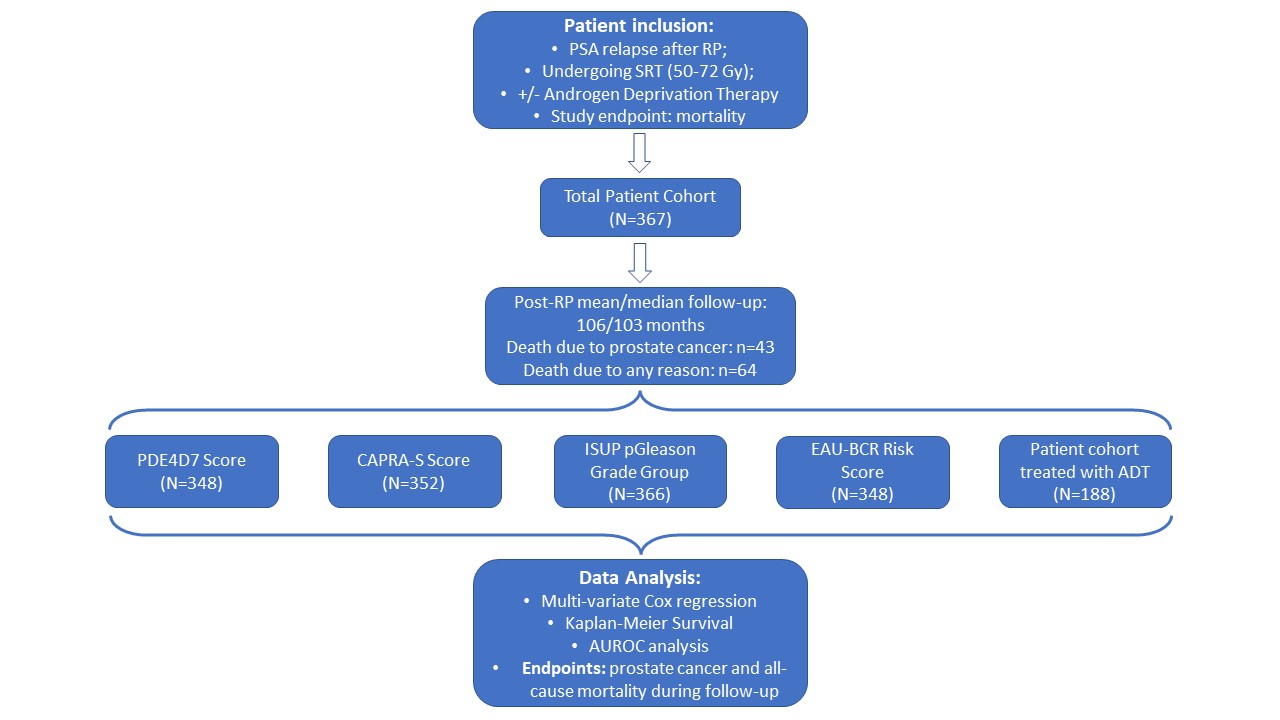
**

**References:**

**CAPRA-S:** Cooperberg MR, Hilton JF, Carroll PR. The CAPRA-S score: a straight-forward tool for improved prediction outcomes after radical prostatectomy. Cancer 2011, 117(22), 5039-5046. https://doi.org/10.1002/cncr.26169

**EAU-BCR Risk:** Tilki D, Preisser F, Graefen M, Huland H, Pompe RS. External Validation of the European Association of Urology Biochemical Recurrence Risk Groups to Predict Metastasis and Mortality After Radical Prostatectomy in a European Cohort. Eur Urol 2019, 75(6), 896-900. https://doi.org/10.1016/j.eururo.2019.03.016

**Supplementary Table 1A:** **Multivariable Cox regression analysis of the PDE4D7 Score.** Multivariable Cox regression analysis of the PDE4D7 Score with the clinical prognostic parameters and the genomic signature mxpGPS (see references) to predict prostate cancer specific death after radical prostatectomy. ISUP Gleason Grade Group was used as integer values 1 to 5. The clinical variables that were used in the model are indicated. pT stage was used as categorical variable (reference: pT2). For the other clinical variables, we used dichotomous inputs into the regression modeling (no = 0; yes = 1).

| **N=315 Patients** | **Multivariable** | | |
| --- | --- | --- | --- |
| **Variable** | **HR** | **95% CI** | **P** |
| **PDE4D7 Score** | 0.52 | 0.31 to 0.87 | 0.01 |
| **mxGPS** | 1.3 | 1.02 to 1.6 | 0.03 |
| **ISUP pGleason Grade Group** | 1.6 | 1.18 to 2.2 | 0.002 |
| **pT_stage="pT3a"** | 0.46 | 0.13 to 1.6 | 0.23 |
| **pT_stage="pT3b"** | 0.2 | 0.03 to 1.1 | 0.07 |
| **pT_stage="pT3c"** | 0.04 | 0 to 0.55 | 0.02 |
| **pT_stage="pT4"** | 0.32 | 0.03 to 3.1 | 0.33 |
| **Extra-Prostatic Extension** | 0.87 | 0.22 to 3.4 | 0.84 |
| **Seminal Vesicle Invasion** | 6.6 | 1.00 to 42.9 | 0.05 |
| **Surgical Margin Status** | 0.42 | 0.19 to 0.89 | 0.02 |
| **Lymph Node Invasion** | 2.4 | 1.06 to 5.2 | 0.03 |

**References:**

**PDE4D7 Score:** Alves de Inda M, van Strijp D, den Biezen-Timmermans E, van Brussel A, Wrobel J, van Zon H, et al. Validation of Cyclic Adenosine Monophosphate Phosphodiesterase-4D7 for its Independent Contribution to Risk Stratification in a Prostate Cancer Patient Cohort with Longitudinal Biological Outcomes. Eur Urol Focus. 2018, 4(3), 376-384. <https://doi.org/10.1016/j.euf.2017.05.010>

**mxGPS:** Salami SS, Hovelson DH, Kaplan JB, Mathieu R, Udager AM, Curci NE, et al. Transcriptomic heterogeneity in multifocal prostate cancer. JCI insight. 2018;3(21):1–13. https://doi.org/10.1172/jci.insight.123468

**Supplementary Table 1B: Multivariable Cox regression analysis of the PDE4D7 Score.** Multivariable Cox regression analysis of the PDE4D7 Score with the clinical prognostic parameters and the genomic signature mxpCCP (see references) to predict prostate cancer specific death after radical prostatectomy. ISUP Gleason Grade Group was used as integer values 1 to 5. The clinical variables that were used in the model are indicated. pT stage was used as categorical variable (reference: pT2). For the other clinical variables, we used dichotomous inputs into the regression modeling (no = 0; yes = 1).

| **N=315 Patients** | **Multivariable** | | |
| --- | --- | --- | --- |
| **Variable** | **HR** | **95% CI** | **P** |
| **PDE4D7 Score** | 0.5 | 0.29 to 0.82 | 0.007 |
| **mxCCP** | 1.3 | 0.97 to 1.7 | 0.08 |
| **ISUP pGleason Grade Group** | 1.7 | 1.3 to 2.3 | 0.0001 |
| **pT_stage="pT3a"** | 0.42 | 0.12 to 1.5 | 0.18 |
| **pT_stage="pT3b"** | 0.18 | 0.03 to 0.99 | 0.05 |
| **pT_stage="pT3c"** | 0.04 | 0.002 to 0.51 | 0.01 |
| **pT_stage="pT4"** | 0.5 | 0.05 to 4.4 | 0.51 |
| **Extra-Prostatic Extension** | 1 | 0.26 to 3.9 | 0.98 |
| **Seminal Vesicle Invasion** | 6.9 | 1.1 to 44.2 | 0.04 |
| **Surgical Margin Status** | 0.41 | 0.19 to 0.88 | 0.02 |
| **Lymph Node Invasion** | 3.1 | 1.4 to 6.6 | 0.005 |

**References:**

**PDE4D7 Score:** Alves de Inda M, van Strijp D, den Biezen-Timmermans E, van Brussel A, Wrobel J, van Zon H, et al. Validation of Cyclic Adenosine Monophosphate Phosphodiesterase-4D7 for its Independent Contribution to Risk Stratification in a Prostate Cancer Patient Cohort with Longitudinal Biological Outcomes. Eur Urol Focus. 2018, 4(3), 376-384. <https://doi.org/10.1016/j.euf.2017.05.010>

**mxpCCP:** Salami SS, Hovelson DH, Kaplan JB, Mathieu R, Udager AM, Curci NE, et al. Transcriptomic heterogeneity in multifocal prostate cancer. JCI insight. 2018;3(21):1–13. https://doi.org/10.1172/jci.insight.123468

**Supplementary Table 1C:** **Multivariable Cox regression analysis of the PDE4D7 Score with the CAPRA-S and the EAU-BCR risk.** Multivariable Cox regression analysis of the PDE4D7 Score with the two clinical prognostic models CAPRA-S and the EAU-BCR risk scores to predict prostate cancer specific death after radical prostatectomy.

| **Model 1: N=326 patients** | | **Multivariable** | | |
| --- | --- | --- | --- | --- |
| **Model** | **Variable** | **HR** | **95% CI** | **P** |
| 1 | **PDE4D7 Score** | 0.36 | 0.23 to 0.57 | <0.0001 |
| 1 | **EAU-BCR Risk** | 3.1 | 1.4 to 6.5 | 0.004 |
| **Model 2: N=330 patients** | | **Multivariable** | | |
| 2 | **PDE4D7 Score** | 0.31 | 0.2 to 0.48 | <0.0001 |
| 2 | **CAPRA-S Score** | 1.1 | 0.95 to 1.2 | 0.2 |

**References:**

**PDE4D7 Score:** Alves de Inda M, van Strijp D, den Biezen-Timmermans E, van Brussel A, Wrobel J, van Zon H, et al. Validation of Cyclic Adenosine Monophosphate Phosphodiesterase-4D7 for its Independent Contribution to Risk Stratification in a Prostate Cancer Patient Cohort with Longitudinal Biological Outcomes. Eur Urol Focus. 2018, 4(3), 376-384. <https://doi.org/10.1016/j.euf.2017.05.010>

**CPARA-S:** Cooperberg MR, Hilton JF, Carroll PR. The CAPRA-S score: a straight-forward tool for improved prediction outcomes after radical prostatectomy. Cancer 2011, 117(22), 5039-5046. https://doi.org/10.1002/cncr.26169

**EAU-BCR Risk:** Tilki D, Preisser F, Graefen M, Huland H, Pompe RS. External Validation of the European Association of Urology Biochemical Recurrence Risk Groups to Predict Metastasis and Mortality After Radical Prostatectomy in a European Cohort. Eur Urol 2019, 75(6), 896-900. https://doi.org/10.1016/j.eururo.2019.03.016

**Supplementary Table 1D: AUROC analysis to predict 5-year post-SRT prostate cancer specific mortality.** Univariable logistic regression analysis of the PDE4D7 Score with the clinical prognostic parameters and the genomic signature mxpGPS (see references) and the two clinical models CAPRA-S and the EAU-BCR Risk score to predict 5-year prostate cancer specific death after radical prostatectomy. **PDE4D7_EAU-BCR model:** combination logistic regression risk model to combine the PDE4D7 and the EAU Risk scores. **PDE4D7_Full_Clinical model:** combination logistic regression risk model to combine the PDE4D7 score with ISUP Gleason grade group, pT stage, extra-prostatic extension, seminal vesicle invasion, surgical margin status, and lymph node invasion (see also Supplementary Table 2). **PDE4D7_mxpGPS model:** combination logistic regression risk model to combine the PDE4D7 score with the genomic mxpGPS risk score.

| **Variable** | **AUROC** | **P** | **N** | **Events (%)** |
| --- | --- | --- | --- | --- |
| **PDE4D7 Score** | 0.77 | <0.0001 | 240 | 20 (8.3) |
| **mxpGPS** | 0.77 | 0.0006 | 236 | 19 (8.1) |
| **ISUP Gleason Grade Group** | 0.69 | 0.003 | 253 | 21 (8.3) |
| **EAU-BCR Risk Score** | 0.68 | 0.004 | 237 | 21 (8.9) |
| **CAPRA-S Score** | 0.58 | 0.25 | 241 | 21 (8.7) |
| **PDE4D7_EAU-BCR Model** | 0.81 | <0.0001 | 223 | 20 (9.0) |
| **PDE4D7_Full_Clinical Model** | 0.88 | <0.0001 | 235 | 20 (8.5) |
| **PDE4D7_mxpGPS Model** | 0.81 | <0.0001 | 223 | 18 (8.1) |

**References:**

**PDE4D7 Score:** Alves de Inda M, van Strijp D, den Biezen-Timmermans E, van Brussel A, Wrobel J, van Zon H, et al. Validation of Cyclic Adenosine Monophosphate Phosphodiesterase-4D7 for its Independent Contribution to Risk Stratification in a Prostate Cancer Patient Cohort with Longitudinal Biological Outcomes. Eur Urol Focus. 2018, 4(3), 376-384. <https://doi.org/10.1016/j.euf.2017.05.010>

**mxpGPS:** Salami SS, Hovelson DH, Kaplan JB, Mathieu R, Udager AM, Curci NE, et al. Transcriptomic heterogeneity in multifocal prostate cancer. JCI insight. 2018;3(21):1–13. https://doi.org/10.1172/jci.insight.123468

**Supplementary Figure 2A: Kaplan Meier survival analysis of the mxpGPS Risk Score.** Kaplan Meier survival analysis of the mxpGPS Risk Score to prostate cancer specific mortality (PCSM) after start of androgen deprivation therapy (ADT) in additional to the applied pelvic radiation treatment. We used a cut-off for the mxpGPS which was used before in previous studies (see references). The residual patients at risk for each 40 months after ADT start are indicated. The logrank p-value to stratify the patients into two groups with different survival probability is given.

**
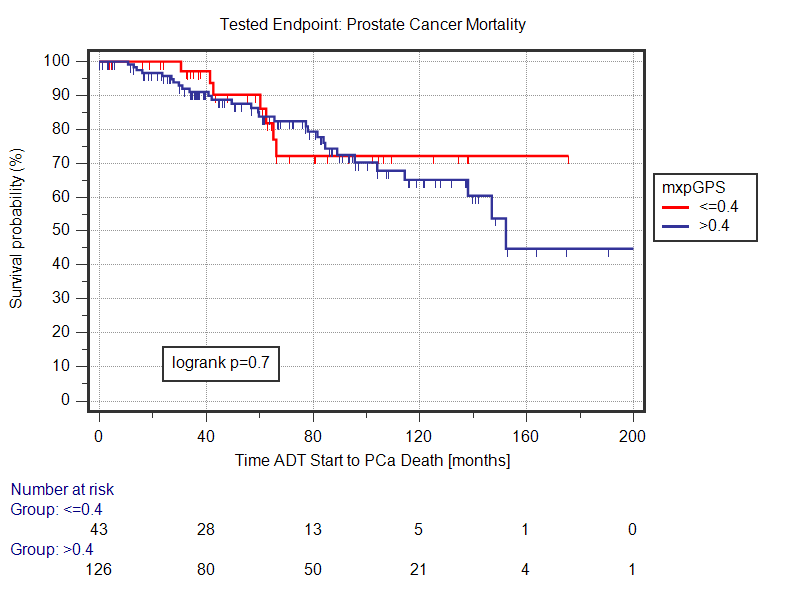
**

**References:**

**mxpGPS:** Salami SS, Hovelson DH, Kaplan JB, Mathieu R, Udager AM, Curci NE, et al. Transcriptomic heterogeneity in multifocal prostate cancer. JCI insight. 2018;3(21):1–13. https://doi.org/10.1172/jci.insight.123468

**Supplementary Figure 2B: Kaplan Meier survival analysis of the EAU-BCR Risk Score.** Kaplan Meier survival analysis of the EAU-BCR Risk score to prostate cancer specific mortality (PCSM) after start of androgen deprivation therapy (ADT) in additional to the applied pelvic radiation treatment. We used a cut-off for the EAU-BCR Risk score which was published before in previous studies (see references). The residual patients at risk for each 40 months after ADT start are indicated. The logrank p-value to stratify the patients into two groups with different survival probability is given.

**
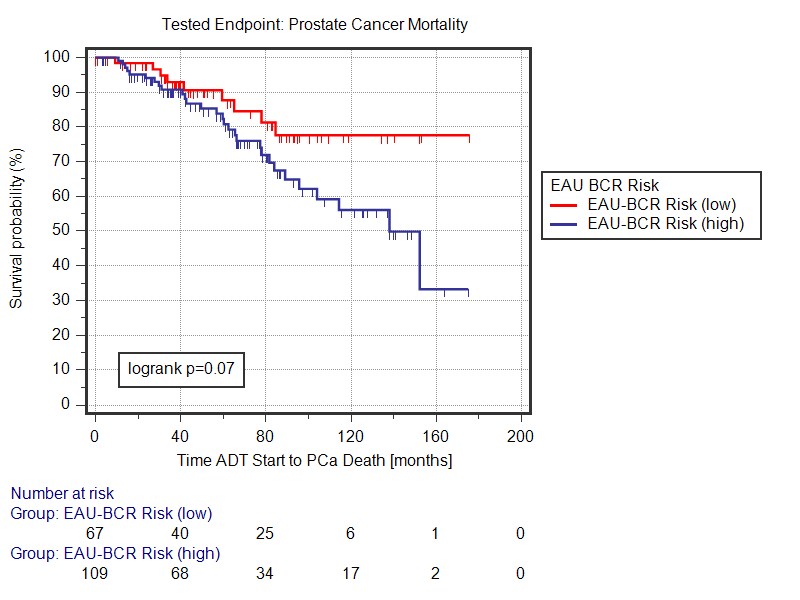
**

**References:**

**EAU-BCR Risk:** Tilki D, Preisser F, Graefen M, Huland H, Pompe RS. External Validation of the European Association of Urology Biochemical Recurrence Risk Groups to Predict Metastasis and Mortality After Radical Prostatectomy in a European Cohort. Eur Urol 2019, 75(6), 896-900. https://doi.org/10.1016/j.eururo.2019.03.016

**Supplementary Figure 2C: Kaplan Meier survival analysis of the CAPRA-S Risk Score.** Kaplan Meier survival analysis of the CAPRA-S Risk Score to prostate cancer specific mortality (PCSM) after start of androgen deprivation therapy (ADT) in additional to the applied pelvic radiation treatment. We categorized patients into three risk classes as previously suggested (see references). The residual patients at risk for each 40 months after ADT start are indicated. The logrank p-value to stratify the patients into two groups with different survival probability is given.

**
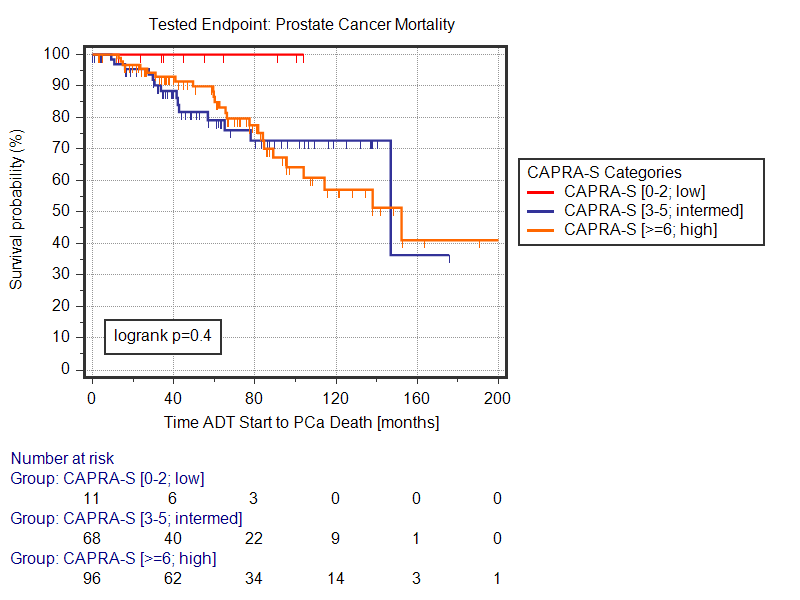
**

**References:**

**CPARA-S:** Cooperberg MR, Hilton JF, Carroll PR. The CAPRA-S score: a straight-forward tool for improved predicion outcomes after radical prostatectomy. Cancer 2011, 117(22), 5039-5046. https://doi.org/10.1002/cncr.26169

**Supplementary Figure 3A: Lentivirus-mediated shRNA strategy for selective knockdown of PDE4D7.** The figure illustrates the plasmid map of the used lentivirus to stably transfect LNCaP FCG wildtype cells. The vector includes the structural element of a short hairpin shRNA which targets the first coding exon of the PDE4D7 transcript, and its expression is driven by the H1 promoter.


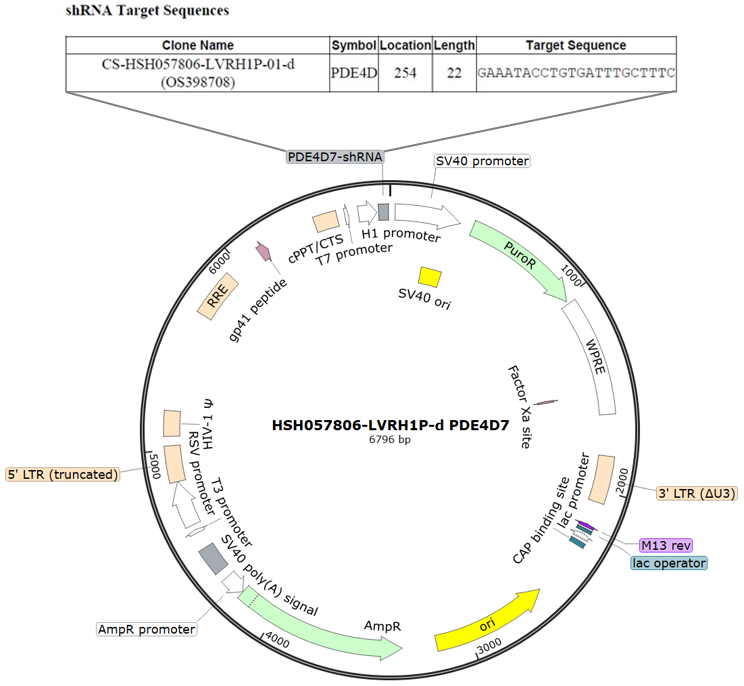


**Supplementary Figure 3B: Lentivirus-mediated Tet-On strategy for inducible PDE4D7 expression.** The figure illustrates the plasmid map of the used lentivirus to stably transfect LNCaP FCG PDE4D7 shRNA knock-down cells. In Tet-On system, the target gene expression is initiated when rtTA (reverse tetracycline-controlled transactivator) is enabled to bind to tet promoter (part of the tet operator) in the presence of tetracycline or its analogues (doxycycline).

**
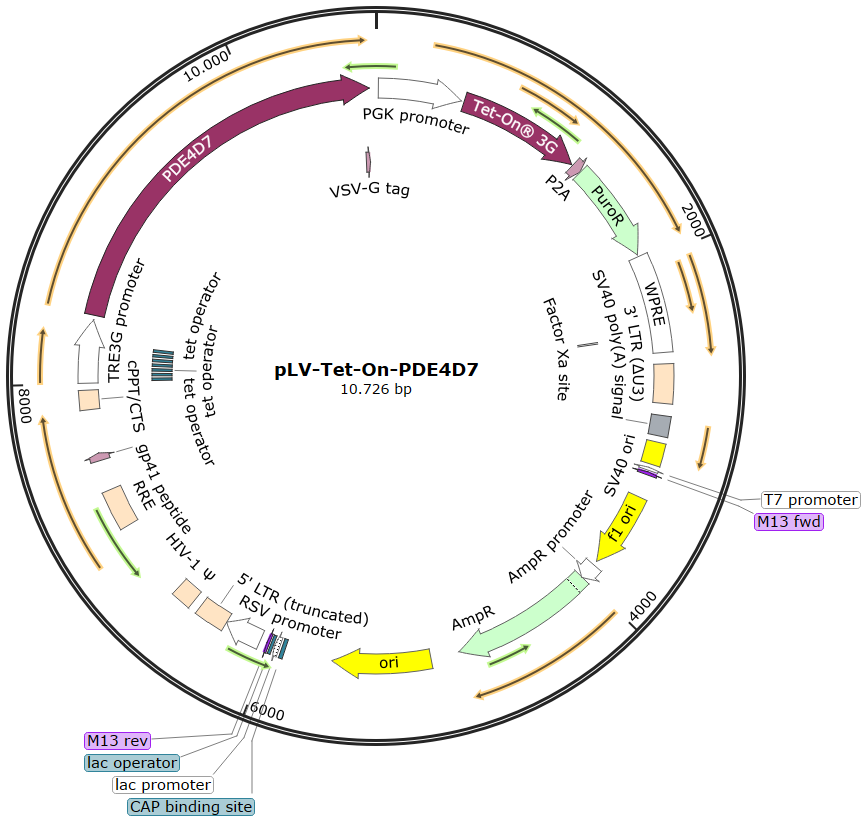
**

**Supplementary Figure 4A: Difference in PDE4D5 mRNA expression between knockdown LNCaP cell line clones.**

**Supplementary Figure 4B: Difference in PDE4D9 mRNA expression between knockdown LNCaP cell line clones.**

**Supplementary Figure 4C:** PDE4D7 protein expression in LNCaP FGC wild-type (WT) and shRNA-PDE4D7 knockdown (P1 and w6.3) LNCaP cell lines

**Supplementary Figure 4D:** Stable knockdown of PDE4D7 in LNCaP cell line clones (P1 and w5.2) enhances real-time growth in comparison to LNCaP wild-type (WT) cells

**Supplementary Figure 4E:** Stable knockdown of PDE4D7 in LNCaP cell line clones (P1 and w6.3) enhances real-time growth in comparison to LNCaP wild-type (WT) cells

**Supplementary Figure 4:** (A & B) RT-qPCR of knockdown LNCaP cell lines shows fold changes in gene expression relative to WT (2^-∆∆Ct^) for PDE4D5 (A) and PDE4D9 (B). One-way ANOVA was performed on ∆∆Ct values. C) PDE4D7 protein expression in LNCaP WT and shRNA-PDE4D7 (P1 and w6.3) LNCaPs. Quantified to total protein stain (TPS) and statistical analysis via one-way ANOVA. (D & E) Real-time proliferation of LNCaP WT and shRNA-PDE4D7 clone P1 vs shRNA-PDE4D7 clone w5.2 (D) and shRNA-PDE4D7 clone w6.3 (E). Slope analysis 24-48 h post-seeding. All error bars represent mean +/- SEM for N=3 (* p<0.05, ** p<0.01, **** p<0.0001, ns = non-significant.)

**Supplementary Table 2: Gene Set Enrichment Analysis (www.gsea-msigdb.org) of Hallmark Pathways (1).** Shown are the top 20 Hallmark Pathways depleted in P1, w5.2, and w6.3 vs. LNCaP wild type and scrambled SC2 control after GSEA. **SIZE** – number of genes in the Hallmark Pathway; **ES** – enrichment score; **NES** – normalized enrichment score; **NOM p-value** – nominal p-value; **FDR q-value** – false discovery rate q-value.

| **NAME** | **SIZE** | **ES** | **NES** | **NOM p-value** | **FDR q-value** |
| --- | --- | --- | --- | --- | --- |
| HALLMARK_ANDROGEN_RESPONSE | 100 | -0.7 | -2.1 | <1.00E-03 | <1.00E-03 |
| HALLMARK_OXIDATIVE_PHOSPHORYLATION | 200 | -0.6 | -1.8 | <1.00E-03 | 1.00E-03 |
| HALLMARK_FATTY_ACID_METABOLISM | 158 | -0.5 | -1.7 | <1.00E-03 | 2.00E-03 |
| HALLMARK_MYC_TARGETS_V2 | 58 | -0.6 | -1.7 | <1.00E-03 | 2.00E-03 |
| HALLMARK_MTORC1_SIGNALING | 200 | -0.5 | -1.6 | <1.00E-03 | 6.00E-03 |
| HALLMARK_REACTIVE_OXYGEN_SPECIES_PATHWAY | 49 | -0.6 | -1.5 | 2.40E-02 | 1.00E-02 |
| HALLMARK_CHOLESTEROL_HOMEOSTASIS | 74 | -0.5 | -1.5 | 3.00E-03 | 1.20E-02 |
| HALLMARK_PEROXISOME | 104 | -0.5 | -1.5 | 6.00E-03 | 1.70E-02 |
| HALLMARK_XENOBIOTIC_METABOLISM | 200 | -0.4 | -1.4 | <1.00E-03 | 2.00E-02 |
| HALLMARK_BILE_ACID_METABOLISM | 112 | -0.5 | -1.4 | 3.00E-03 | 2.10E-02 |
| HALLMARK_ESTROGEN_RESPONSE_LATE | 200 | -0.4 | -1.4 | <1.00E-03 | 3.30E-02 |
| HALLMARK_INTERFERON_ALPHA_RESPONSE | 97 | -0.5 | -1.4 | 1.20E-02 | 3.30E-02 |
| HALLMARK_PI3K_AKT_MTOR_SIGNALING | 105 | -0.4 | -1.3 | 3.40E-02 | 4.80E-02 |
| HALLMARK_UNFOLDED_PROTEIN_RESPONSE | 113 | -0.4 | -1.3 | 3.30E-02 | 5.10E-02 |
| HALLMARK_ADIPOGENESIS | 200 | -0.4 | -1.3 | 1.00E-02 | 5.10E-02 |
| HALLMARK_INTERFERON_GAMMA_RESPONSE | 200 | -0.4 | -1.2 | 4.70E-02 | 8.60E-02 |
| HALLMARK_P53_PATHWAY | 200 | -0.4 | -1.2 | 3.50E-02 | 9.40E-02 |
| HALLMARK_ESTROGEN_RESPONSE_EARLY | 200 | -0.4 | -1.2 | 6.00E-02 | 1.03E-01 |
| HALLMARK_MYC_TARGETS_V1 | 200 | -0.4 | -1.2 | 6.60E-02 | 1.07E-01 |
| HALLMARK_DNA_REPAIR | 150 | -0.3 | -1.1 | 2.12E-01 | 2.57E-01 |

**Supplementary Figure 5: Protein expression of prostate cancer related RNAseq identified gene alterations.** Western blot and quantification of protein expression of PCa-related RNA-seq genes in LNCaP wildtype and scrambled shRNA control cell line. WT – LNCaP FCG wild-type; SC2 – LNCaP scrambled shRNA control (N=3).

**Supplementary Table 3: Gene Set Enrichment Analysis (www.gsea-msigdb.org) of Hallmark Pathways (2).** Top 20 Hallmark Pathways enriched in LNCaP PDE4D7 knock-down cell clones P1, w5.2, and w6.3 vs. LNCaP wild type and scrambled SC2 control after GSEA. **SIZE** – number of genes in the Hallmark Pathway; **ES** – enrichment score; **NES** – normalized enrichment score; **NOM p-value** – nominal p-value; **FDR q-value** – false discovery rate q-value.

| **NAME** | **SIZE** | **ES** | **NES** | **NOM p-val** | **FDR q-val** |
| --- | --- | --- | --- | --- | --- |
| HALLMARK_EPITHELIAL_MESENCHYMAL_TRANSITION | 200 | 0.6 | 1.9 | <1.00E-03 | <1.00E-03 |
| HALLMARK_PANCREAS_BETA_CELLS | 40 | 0.6 | 1.5 | 7.00E-03 | 2.00E-02 |
| HALLMARK_SPERMATOGENESIS | 135 | 0.5 | 1.6 | 1.00E-03 | 2.50E-02 |
| HALLMARK_MITOTIC_SPINDLE | 199 | 0.5 | 1.5 | 1.00E-03 | 4.70E-02 |
| HALLMARK_HYPOXIA | 200 | 0.5 | 1.4 | 3.00E-03 | 5.20E-02 |
| HALLMARK_UV_RESPONSE_DN | 144 | 0.5 | 1.4 | 4.00E-03 | 5.30E-02 |
| HALLMARK_INFLAMMATORY_RESPONSE | 200 | 0.5 | 1.4 | 4.00E-03 | 6.50E-02 |
| HALLMARK_ALLOGRAFT_REJECTION | 200 | 0.5 | 1.4 | 4.00E-03 | 7.30E-02 |
| HALLMARK_COMPLEMENT | 200 | 0.4 | 1.3 | 2.30E-02 | 7.80E-02 |
| HALLMARK_KRAS_SIGNALING_UP | 200 | 0.4 | 1.3 | 1.30E-02 | 7.90E-02 |
| HALLMARK_MYOGENESIS | 200 | 0.4 | 1.3 | 7.00E-03 | 8.70E-02 |
| HALLMARK_HEDGEHOG_SIGNALING | 36 | 0.5 | 1.3 | 1.35E-01 | 9.00E-02 |
| HALLMARK_WNT_BETA_CATENIN_SIGNALING | 42 | 0.5 | 1.3 | 1.01E-01 | 9.40E-02 |
| HALLMARK_IL2_STAT5_SIGNALING | 199 | 0.4 | 1.3 | 1.80E-02 | 9.60E-02 |
| HALLMARK_ANGIOGENESIS | 36 | 0.5 | 1.3 | 1.18E-01 | 1.07E-01 |
| HALLMARK_KRAS_SIGNALING_DN | 200 | 0.4 | 1.3 | 3.80E-02 | 1.12E-01 |
| HALLMARK_TGF_BETA_SIGNALING | 54 | 0.5 | 1.2 | 1.24E-01 | 1.28E-01 |
| HALLMARK_G2M_CHECKPOINT | 200 | 0.4 | 1.2 | 1.18E-01 | 2.41E-01 |
| HALLMARK_APOPTOSIS | 161 | 0.4 | 1.1 | 1.98E-01 | 3.36E-01 |
| HALLMARK_IL6_JAK_STAT3_SIGNALING | 87 | 0.4 | 1.1 | 2.48E-01 | 3.42E-01 |

**Supplementary Table 4:** Overview of genes altered in NEPC (see references)

| **#** | **Type** | **Genes** | **Aberration in t-NEPC** | **Literature** | **Expression in PDE4D7 kd vs control cell lines** | |
| --- | --- | --- | --- | --- | --- | --- |
| 1 | **Genetic** | PTEN | Deletion | 1 | down | |
| 2 |  | TP53 | Mutation | 1 | NA | |
| 3 |  | RB1 | Deletion | 1 | down | |
| 4 |  | AURKA | Amplification | 1,2 | up | |
| 5 |  | MYCN | Amplification | 1,2 | up | |
| 6 | **Transcription factors** | ASCL1 | Induced | 1 | up | |
| 7 |  | FOXA1 | Reduced | 1,2 | down | |
| 8 |  | FOXA2 | Upregulated | 1,2 | up | |
| 9 |  | FOXB2 | Upregulated | 1,2 | down | |
| 10 |  | TTF1 | Upregulated | 1 | up | |
| 11 |  | NKX3-1 | Reduced | 1 | down | |
| 12 |  | REST | Reduced | 1 | down | |
| 13 |  | ONECUT2 | Upregulated | 1,2 | up | |
| 14 |  | POU3F2 | Upregulated | 1,2 | up | |
| 15 |  | POU3F4 | Upregulated | 1 | not expressed | |
| 16 |  | SOX2 | Upregulated | 1,2 | up | |
| 17 |  | ZBTB46 | Upregulated | 1,2 | up | |
| 18 | **Epigenetics** | EZH2 | Upregulated | 1 | up | |
| 19 |  | PHF8 | Upregulated | 1,2 | down | |
| 20 |  | KDM1A | Alternative splicing | 1 | NA | |
| 21 |  | MEAF6 | Alternative splicing | 1 | NA | |
| 22 |  | SMARCA4 | Upregulated | 1 | down | |
| 23 |  | CBX5 | Upregulated | 1 | down | |
| 24 | **DNA repair pathways** | PARP1 | Upregulated | 1,2 | up | |
| 25 |  | SLFN11 | Reduced | 1 | not expressed | |
| 26 | **Other nuclear factors** | CCND1 | Upregulated / Reduced | 1 | down | |
| 27 |  | LIN28B | Upregulated | 1,2 | up | |
| 28 |  | PEG10 | Upregulated | 1 | down | |
| 29 |  | SRRM4 | Upregulated | 1 | down | |
| 30 |  | GIT1 | Alternative splicing | 1 | NA | |
| 31 |  | BIF1 | Alternative splicing | 1 | NA | |
| 32 |  | BHC80 | Alternative splicing | 1 | NA | |
| 33 | **Signaling pathways** | mTOR | Increased activity | 1 | NA | |
| 34 |  | PRKCI | Reduced | 1 | up | |
| 35 |  | STAT3 | Increased activity | 1 | NA | |
| 36 |  | LIFR | Upregulated | 1,2 | up | |
| 37 |  | WNT7B | Upregulated | 1 | down | |
| 38 |  | WNT11 | Upregulated | 1 | up | |
| 39 |  | WLS | Upregulated | 1,2 | up | |
| 40 |  | PCDH11Y | Upregulated | 1 | down | |
| 41 |  | CREB1 | Increased activation | 1 | NA | |
| 42 |  | RET | Upregulated | 1 | up | |
| 43 |  | ETV1 | Reduced | 3 | down | |
| 44 |  | KLK2 | Reduced | 3 | down | |
| 45 |  | KLK3 | Reduced | 3 | down | |
| 46 |  | AR | Reduced | 2 | down | |
| 47 | **Neuroendocrine markers** | ENO2 | Upregulated | 1 | up | |
| 48 |  | NCAM1 | Upregulated | 1 | up | |
| 49 |  | SYP | Upregulated | 1 | up | |
| 50 | **Angiogenesis** | THBS1 | Reduced | 1,2 | down | |
| **References**  1) Merkens et al. J Exp Clin Cancer Res (2022) 41:46; https://doi.org/10.1186/s13046-022-02255-y | | | | | |
| 2) Xie et al. Journal of Cancer Research and Clinical Oncology (2022) 148:1813–1823; https://doi.org/10.1007/s00432-022-04061-7 | | | | | |

**Supplementary Figure 6A: Expression boxplots of AR and significantly down-regulated AR response genes in PDE4D7 score classes in human clinical patient samples.** The expression of each gene is provided after TPM calculation based on the RNAseq count data. The PDE4D7 score classes represent different categories of PDE4D7 expression based on the PDE4D7 score (see references) where PDE4D7_class1 (most left-hand box) represents the lowest PDE4D7 scores (i.e., lowest PDE4D7 expression) while PDE4D7_class4 (most right-hand box) represents the highest PDE4D7 scores (i.e., highest PDE4D7 expression). The number of patients per class are: PDE4D7_class1 (N=13); PDE4D7_class2 (N=134); PDE4D7_class3 (N=301); PDE4D7_class4 (N=85). The median of the expression per group is indicated by the bar within each box. The red cross represents the mean expression value per group. The circles represent outlier expression values. The x crosses represent far-out outliers. The p-values were calculated by use of ANOVA Kruskal-Wallis test and represent a significant change in expression over the four PDE4D7 classes.


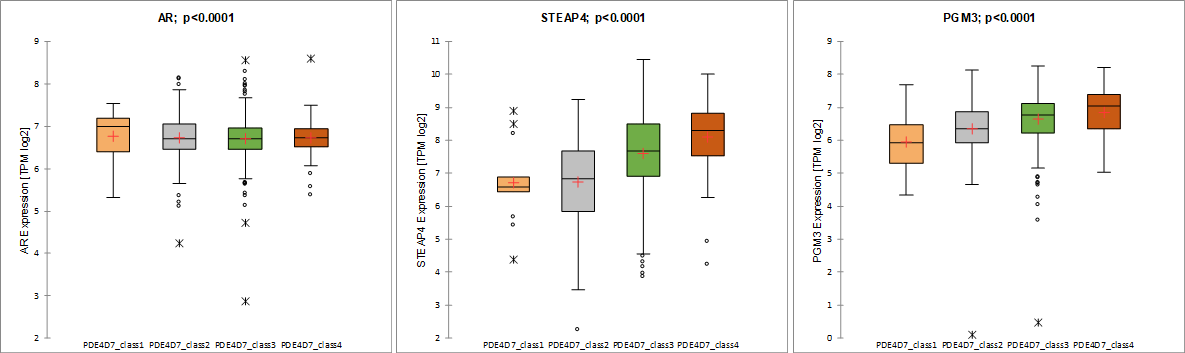


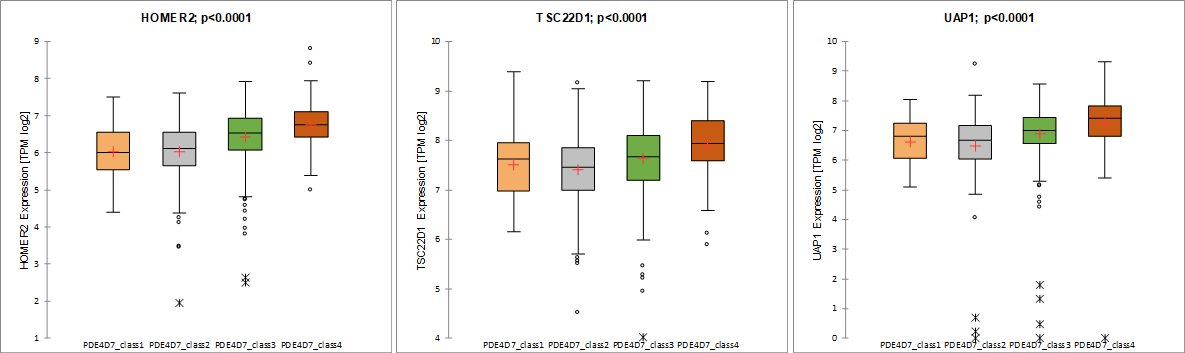


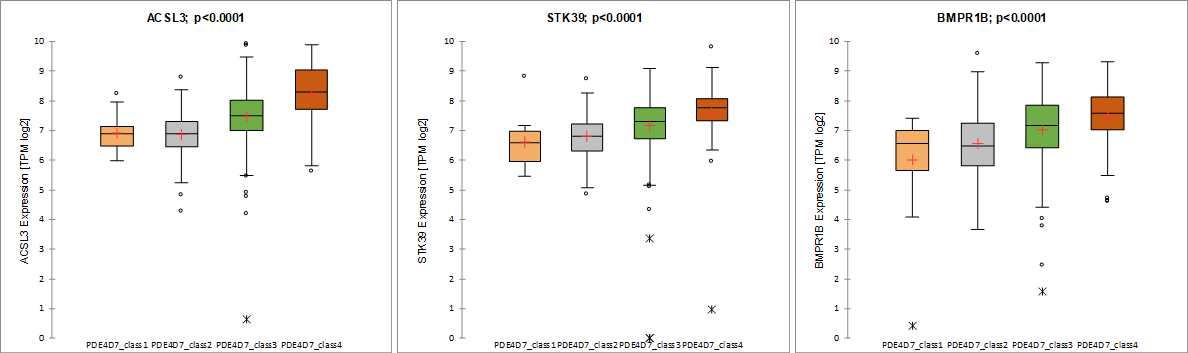


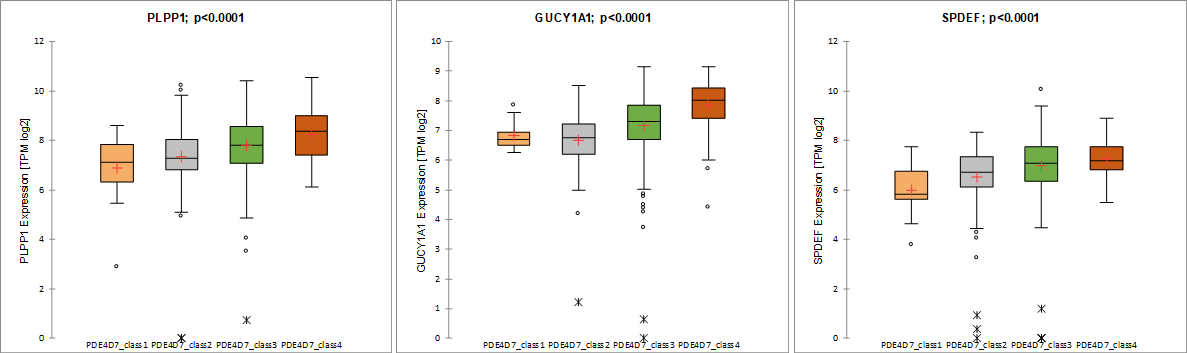


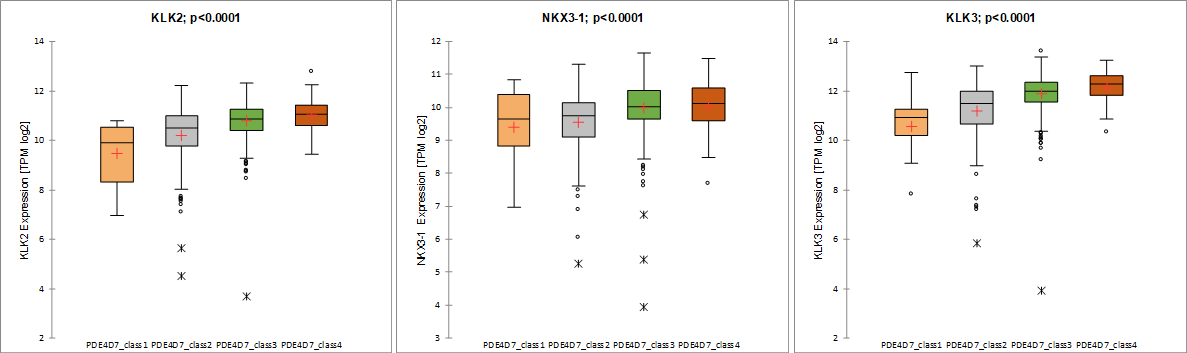


**References:**

Van Strijp D, De Witz C, Heitkötter B, Huss S, Bögemann M, Baillie GS, et al. The association of the long prostate cancer expressed PDE4D transcripts to poor patient outcome depends on the tumour’s TMPRSS2-ERG fusion status. Prostate Cancer. 2019;2019. https://doi.org/10.1155/2019/8107807

**Supplementary Figure 6B: Expression boxplots of genes involved in neuroendocrine differentiation (NED) significantly altered in PDE4D7 score classes in human clinical patient samples.** The expression of each gene is provided after TPM calculation based on the RNAseq count data. The PDE4D7 score classes represent different categories of PDE4D7 expression based on the PDE4D7 score (see references) where PDE4D7_class1 (most left-hand box) represents the lowest PDE4D7 scores (i.e., lowest PDE4D7 expression) while PDE4D7_class4 (most right-hand box) represents the highest PDE4D7 scores (i.e., highest PDE4D7 expression). The number of patients per class are: PDE4D7_class1 (N=13); PDE4D7_class2 (N=134); PDE4D7_class3 (N=301); PDE4D7_class4 (N=85). The median of the expression per group is indicated by the bar within each box. The red cross represents the mean expression value per group. The circles represent outlier expression values. The x crosses represent far-out outliers. The p-values were calculated by use of ANOVA Kruskal-Wallis test and represent a significant change in expression over the four PDE4D7 classes.


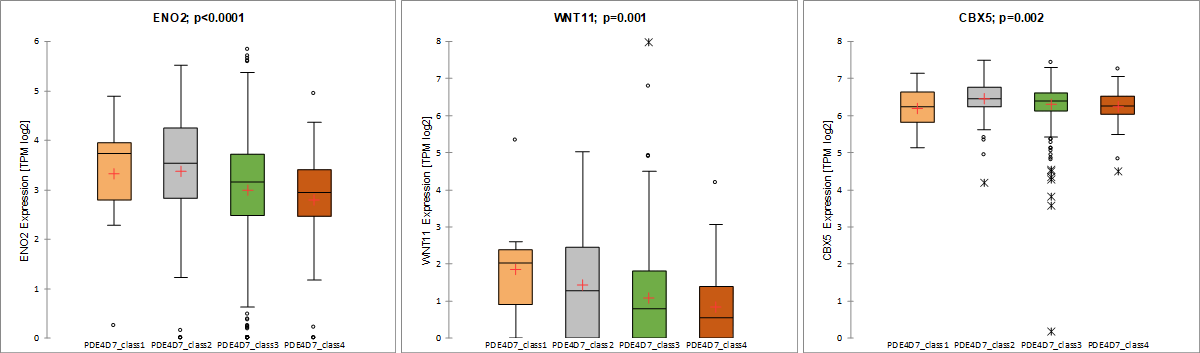


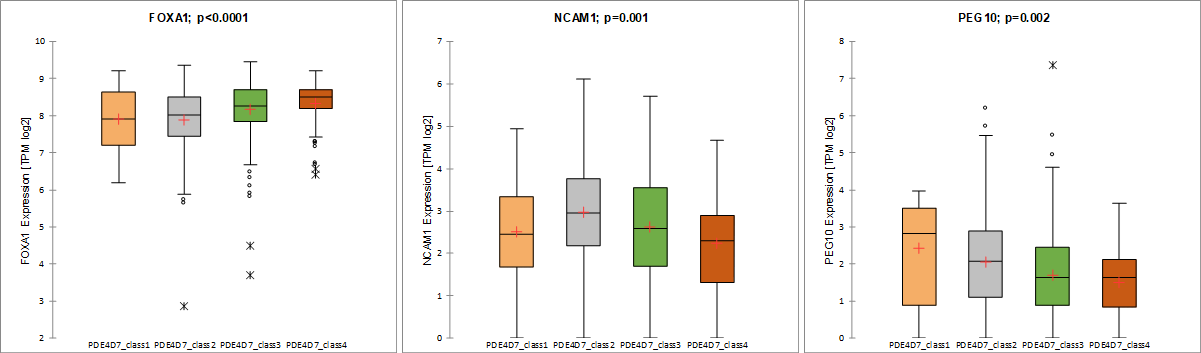


**References:**

Van Strijp D, De Witz C, Heitkötter B, Huss S, Bögemann M, Baillie GS, et al. The association of the long prostate cancer expressed PDE4D transcripts to poor patient outcome depends on the tumour’s TMPRSS2-ERG fusion status. Prostate Cancer. 2019;2019. <https://doi.org/10.1155/2019/8107807>

**Supplementary Table 5:** REACTOME homology-directed repair of DNA double strand breaks gene set (reactome.org: R-HSA-5693538) expanded with additional genes (in red) from other DNA repair pathways known to be altered in prostate cancer (see reference)

| **Gene Symbol** | |
| --- | --- |
| ABRAXAS1 | PPP4C |
| ATM | RAD1 |
| BABAM2 | RAD51C |
| BARD1 | RAD51D |
| BLM | RBBP8 |
| BRCA1 | RFC2 |
| BRCA2 | RFC5 |
| BRIP1 | RMI1 |
| CLSPN | RMI2 |
| DNA2 | RNF168 |
| EME1 | RNF4 |
| EME2 | RNF8 |
| ERCC4 | RPA1 |
| EXO1 | RPA2 |
| H2AFX | RPS27A |
| HIST1H2BM | SPIDR |
| HIST1H2BF | SUMO1 |
| HIST1H2BG | SUMO2 |
| HIST4H4 | TIPIN |
| HIST1H4D | TOPBP1 |
| HERC2 | TP53BP1 |
| HUS1 | UBB |
| KAT5 | UBC |
| MRE11 | UBE2I |
| NBN | UBE2N |
| PARP1 | XRCC1 |
| POLD1 | XRCC2 |
| POLD2 | MLH1 |
| POLD3 | MSH2 |
| POLE | FANCA |
| POLE2 | FANCD2 |
| POLE4 | MSH6 |

**References:**

Lozano R, Castro E, Aragón IM, Cendón Y, Cattrini C, López-Casas PP, et al. Genetic aberrations in DNA repair pathways: a cornerstone of precision oncology in prostate cancer. Br J Cancer 2021, 124(3), 552-563. https://doi.org/10.1038/s41416-020-01114-x

**Supplementary Figures 7A, 7B: Single Nucleotide Variant (SNV) analysis for some key DNA repair genes (see references) in LNCaP FCG wildtype (E) and the PDE4D7 knockdown cell line LNCaP_P1 (F).** Genome sequencing was done in 4 replicates (rep1 to rep4). The affected gene is indicated as well as the resulting SNV and its predicted impact on the translated protein (uncolored – intermediate impact; red – high impact leading typically to a truncated protein version). The identified genotype (GT) in the sample is shown as 0/1 (heterozygous for the SNV) or 1/1 (homozygous for the SNV). The genotype 0/0 (homozygous for the reference nucleotide variant) is represented by the uncolored fields.

**Supplementary Figure 7A**


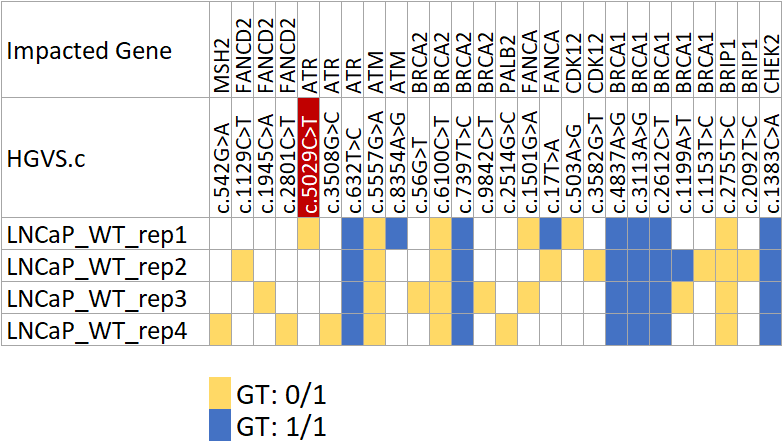


**Supplementary Figure 7B**


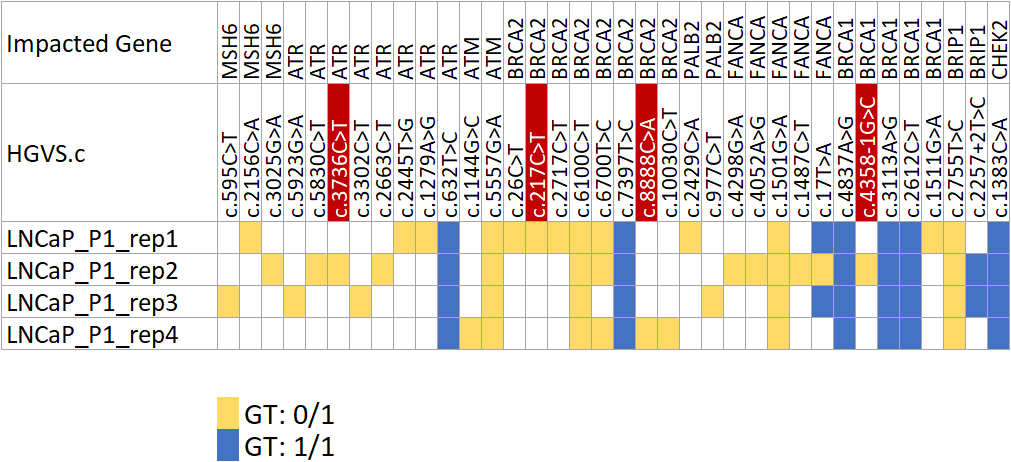


**References**

Cancer Genome Atlas Research Network. The molecular taxonomy of primary prostate cancer. Cell 2015, 163, 1011-1025

Armenia, J., Wankowicz, S. A. M., Liu, D., Gao, J., Kundra, R., Reznik, E. et al. The long tail of oncogenic drivers in prostate cancer. Nat. Genet. **50**, 645–651 (2018)

**Supplementary Figure 8: Expression boxplots of genes involved in homology-directed repair of DNA double strand breaks up-regulated in PDE4D7 score classes in human clinical patient samples.** The expression of each gene is provided after TPM calculation based on the RNAseq count data. The PDE4D7 score classes represent different categories of PDE4D7 expression based on the PDE4D7 score (see references) where PDE4D7_class1 (most left-hand box) represents the lowest PDE4D7 scores (i.e., lowest PDE4D7 expression) while PDE4D7_class4 (most right-hand box) represents the highest PDE4D7 scores (i.e., highest PDE4D7 expression). The number of patients per class are: PDE4D7_class1 (N=13); PDE4D7_class2 (N=134); PDE4D7_class3 (N=301); PDE4D7_class4 (N=85). The median of the expression per group is indicated by the bar within each box. The red cross represents the mean expression value per group. The circles represent outlier expression values. The x crosses represent far-out outliers. The p-values were calculated by use of ANOVA Kruskal-Wallis test and represent a significant change in expression over the four PDE4D7 classes.


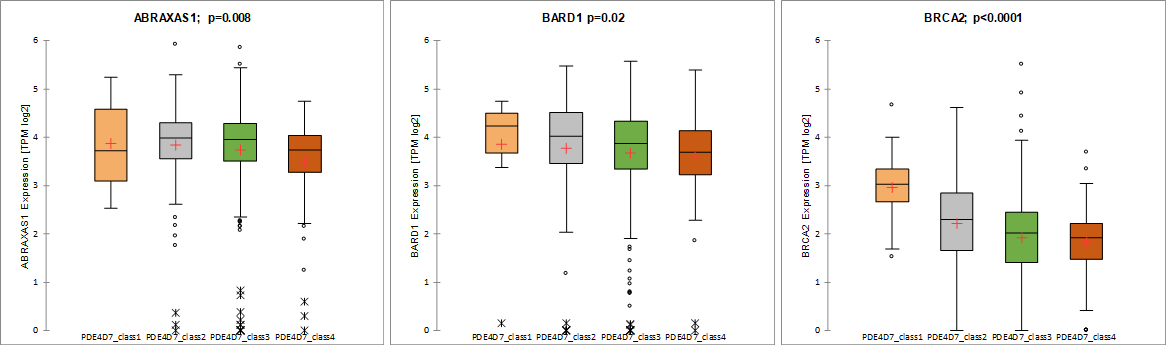


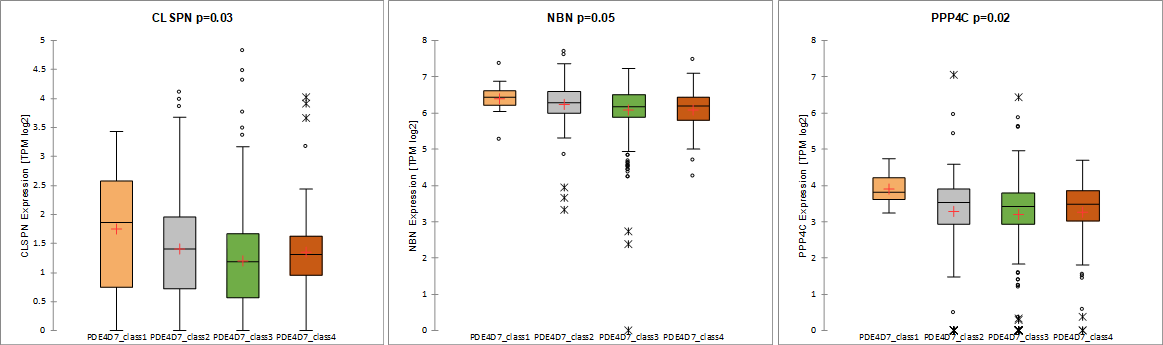


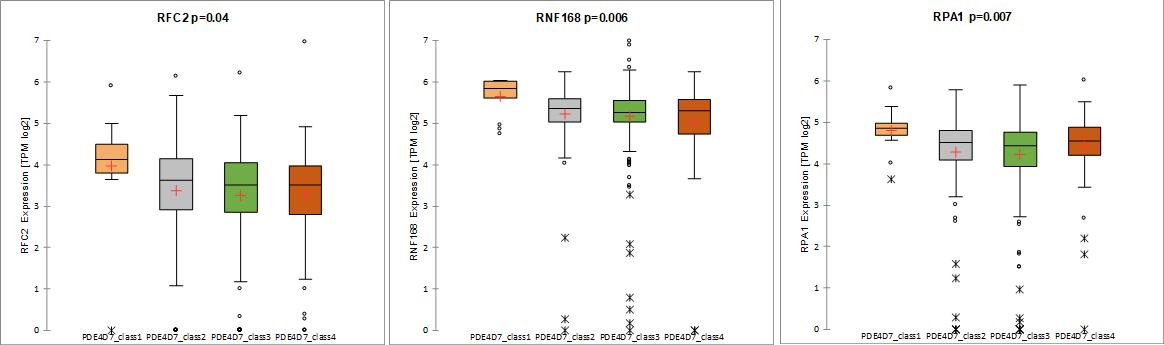


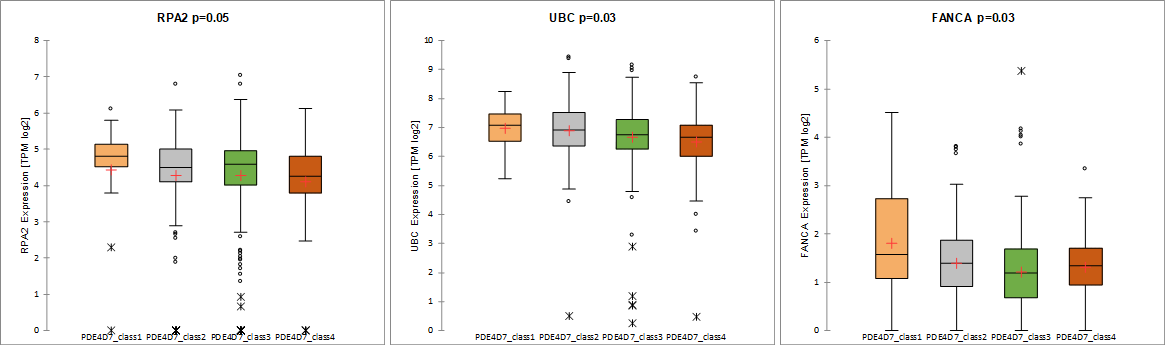


**References:**

Van Strijp D, De Witz C, Heitkötter B, Huss S, Bögemann M, Baillie GS, et al. The association of the long prostate cancer expressed PDE4D transcripts to poor patient outcome depends on the tumour’s TMPRSS2-ERG fusion status. Prostate Cancer. 2019;2019. https://doi.org/10.1155/2019/8107807

**Supplementary Figure 9A-9C: Real-time growth analysis of LNCaP cells with varying PDE4D7 expression upon Docetaxel treatment.** LNCaP FGC wildtype WT **(A)**, LNCaP PDE4D7 knockdown P1 **(B)** growth response to treatment with DNA-damage inducing Docetaxel. Slope analysis 0-48h since treatment. One-way ANOVA was performed on slope values (N=3, * = p≤0.05, ** = p≤0.01, *** = p≤0.001). **C)** Real-time growth of Docetaxel treatment on P1 LNCaP cells re-expressing PDE4D7 (LNCaP-P1- PDE4D7^+^). Slope analysis 0-72h since treatment, and statistical analysis via un-paired t-test (N=3, **** = p≤0.0001).

**Supplementary Figure 9A:** DNA damage induction via Docetaxel affects LNCaP wildtype cells.

**Supplementary Figure 9B:** DNA damage induction via Docetaxel affects LNCaP PDE4D7-knockdown cells

**Supplementary Figure 9C:** Re-expression of PDE4D7 in knockdown LNCaP cell line heightens sensitivity to Docetaxel

**Supplementary Figure 10A: Kaplan Meier survival analysis of the pPDE4D7 Score in ERG fusion negative patient tumours.** Kaplan Meier survival analysis of the pPDE4D7 Score to prostate cancer specific mortality (PCSM) after start of androgen deprivation therapy (ADT) in addition to the applied pelvic radiation treatment (SRT). The pPDE4D7 scores and the respective cut-offs to create the three pPDE4D7 classes were calculated as described in the main text of this publication. The residual patients at risk for each 40 months after ADT start are indicated. The logrank p-value to stratify the patients into two groups with different survival probability is given.

**
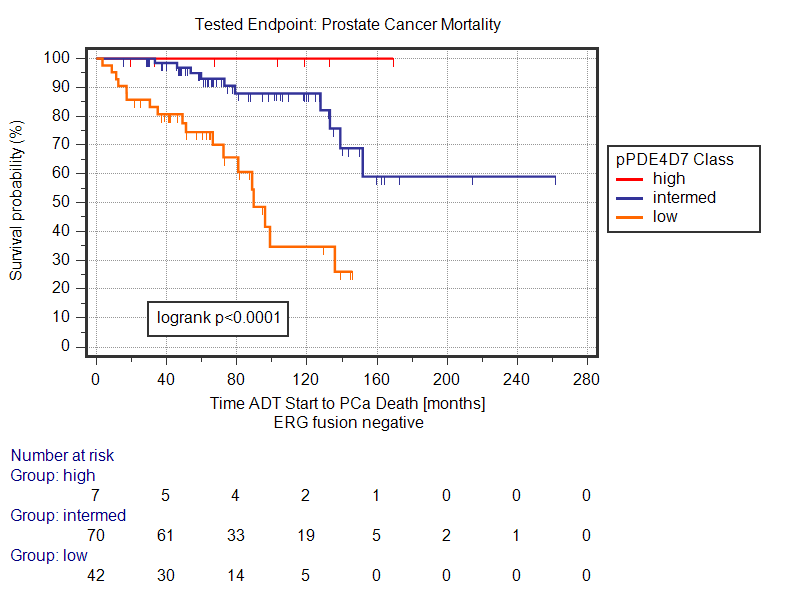
**

**Supplementary Figure 10B: Kaplan Meier survival analysis of the pPDE4D7 Score in ERG fusion positive patient tumours.** Kaplan Meier survival analysis of the pPDE4D7 Score to prostate cancer specific mortality (PCSM) after start of androgen deprivation therapy (ADT) in addition to the applied pelvic radiation treatment (SRT). The pPDE4D7 scores and the respective cut-offs to create the three pPDE4D7 classes were calculated as described in the main text of this publication. The residual patients at risk for each 40 months after ADT start are indicated. The logrank p-value to stratify the patients into two groups with different survival probability is given.

**
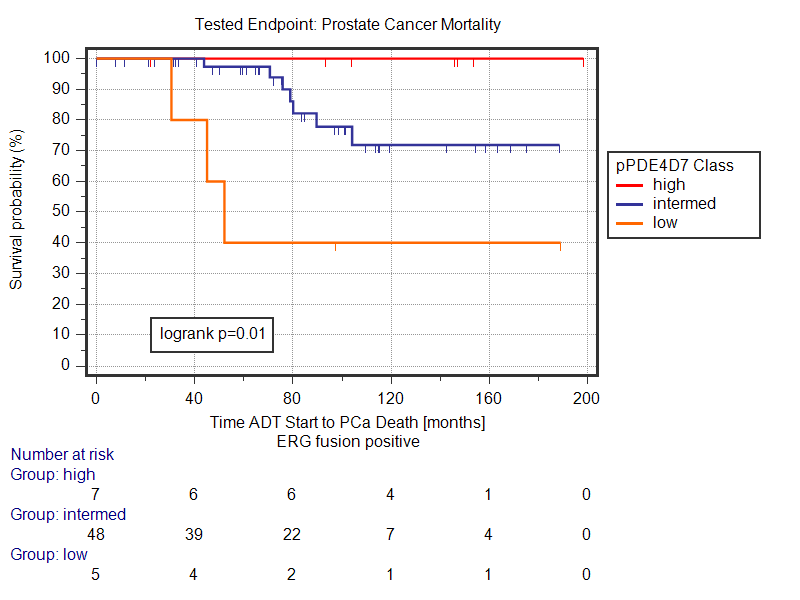
**

**Supplementary Figure 11A-11D: Structural and copy number changes in PDE4D7 knock-down compared to the reference cell lines LNCaP wildtype and LNCaP shRNA scrambled.** **(F)** Structural changes illustrated per chromosome in LNCaP wildtype cell line compared to the human reference genome. Each of the chromosomes is shown individually. Green areas represent chromosomal copy number that are equivalent to the human reference genome. Blue and red indicate areas of copy number losses and copy number gains, respectively. **(G)** Structural changes illustrated per chromosome in LNCaP shRNA scrambled control cell line compared to the human reference genome. The red triangles indicate positions of putative shRNA lentivirus integration into the cellular genome. **(H, I)** Structural changes illustrated per chromosome in LNCaP PDE4D7 knock-down cell lines P1 and w5.2.

**Supplementary Figure 11A:** Structural and copy number changes in LNCaP FGC wildtype

**
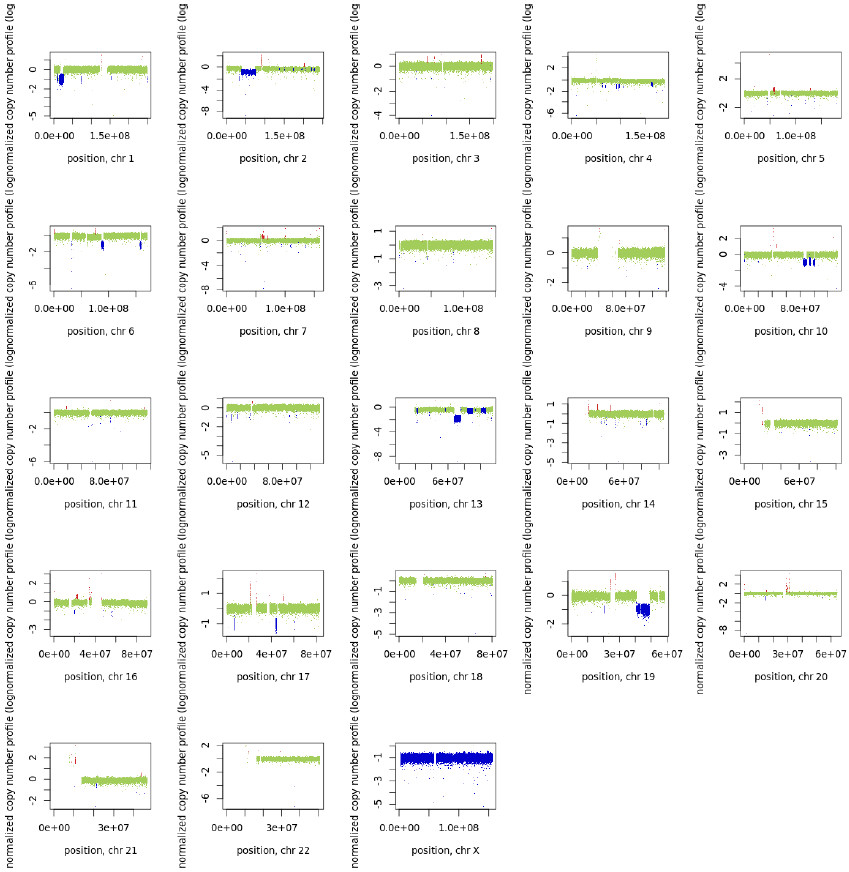
**

**Supplementary Figure 11B:** Structural and copy number changes in LNCaP wildtype scrambled control (SC2)


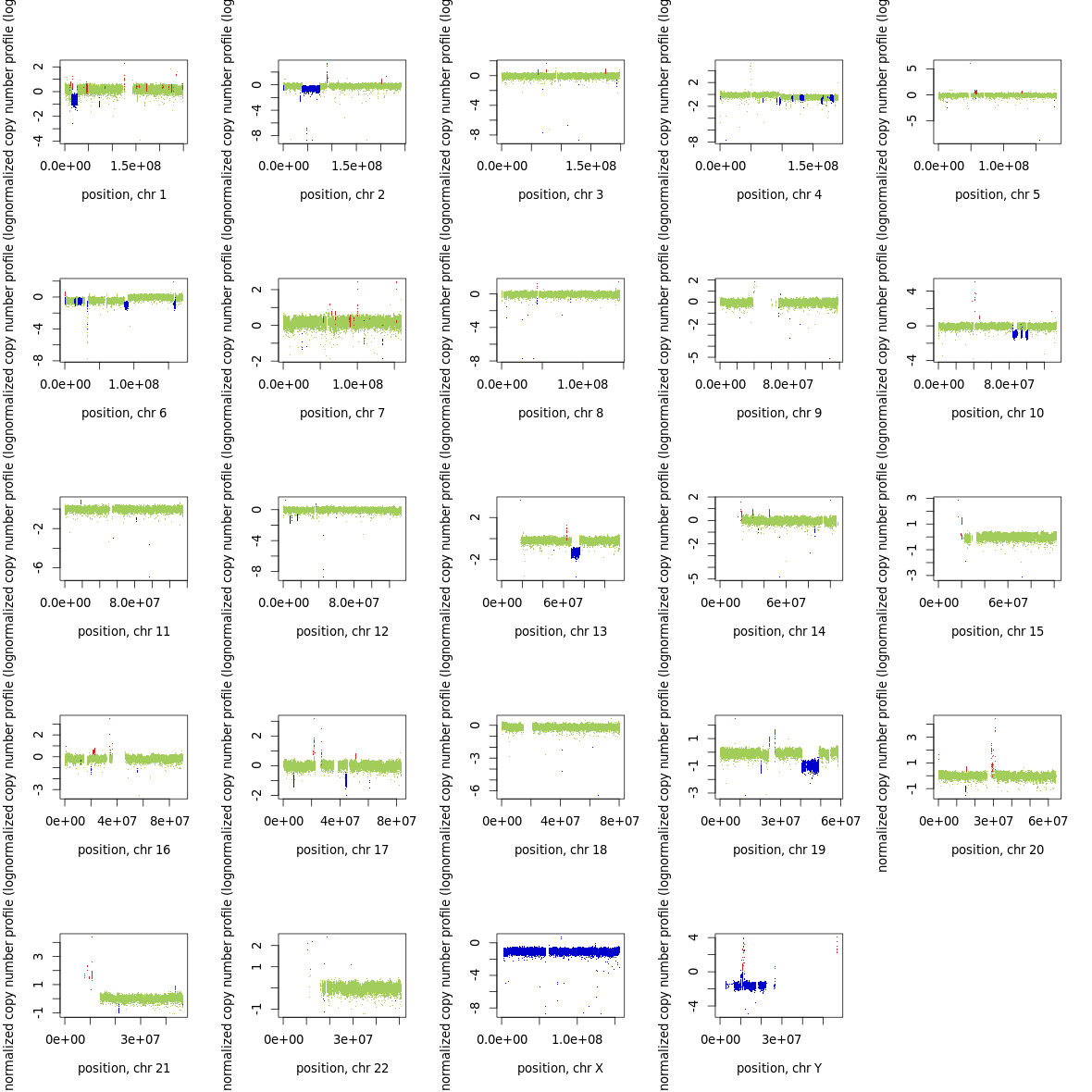


**Figure 8K:**

**Figure 8L:**

**Supplementary Figure 11C:** Structural and copy number changes in LNCaP PDE4D7 knockdown cell line (clone P1)


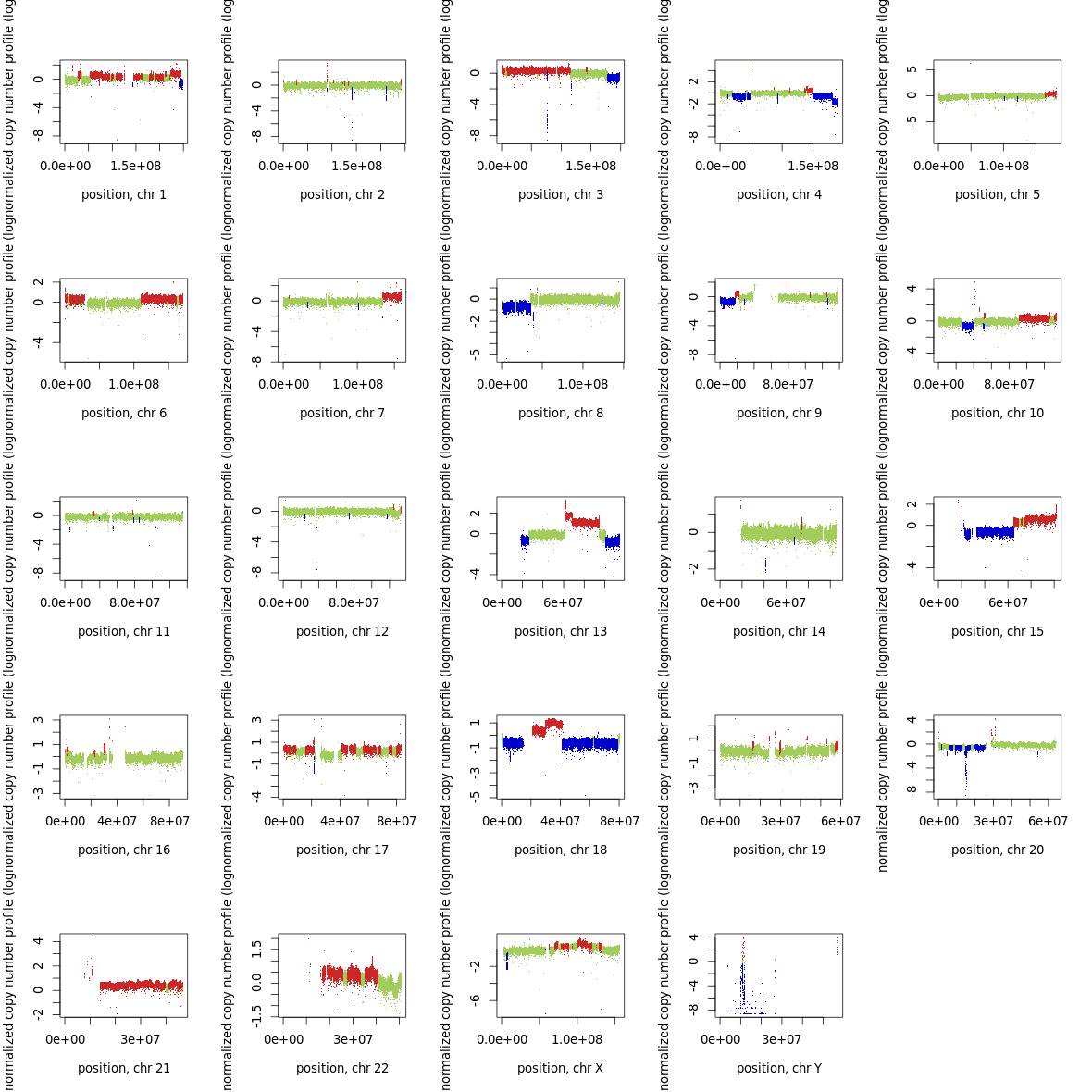


**Supplementary Figure 11D:** Structural and copy number changes in LNCaP PDE4D7 knockdown cell line (clone w5.2)

**
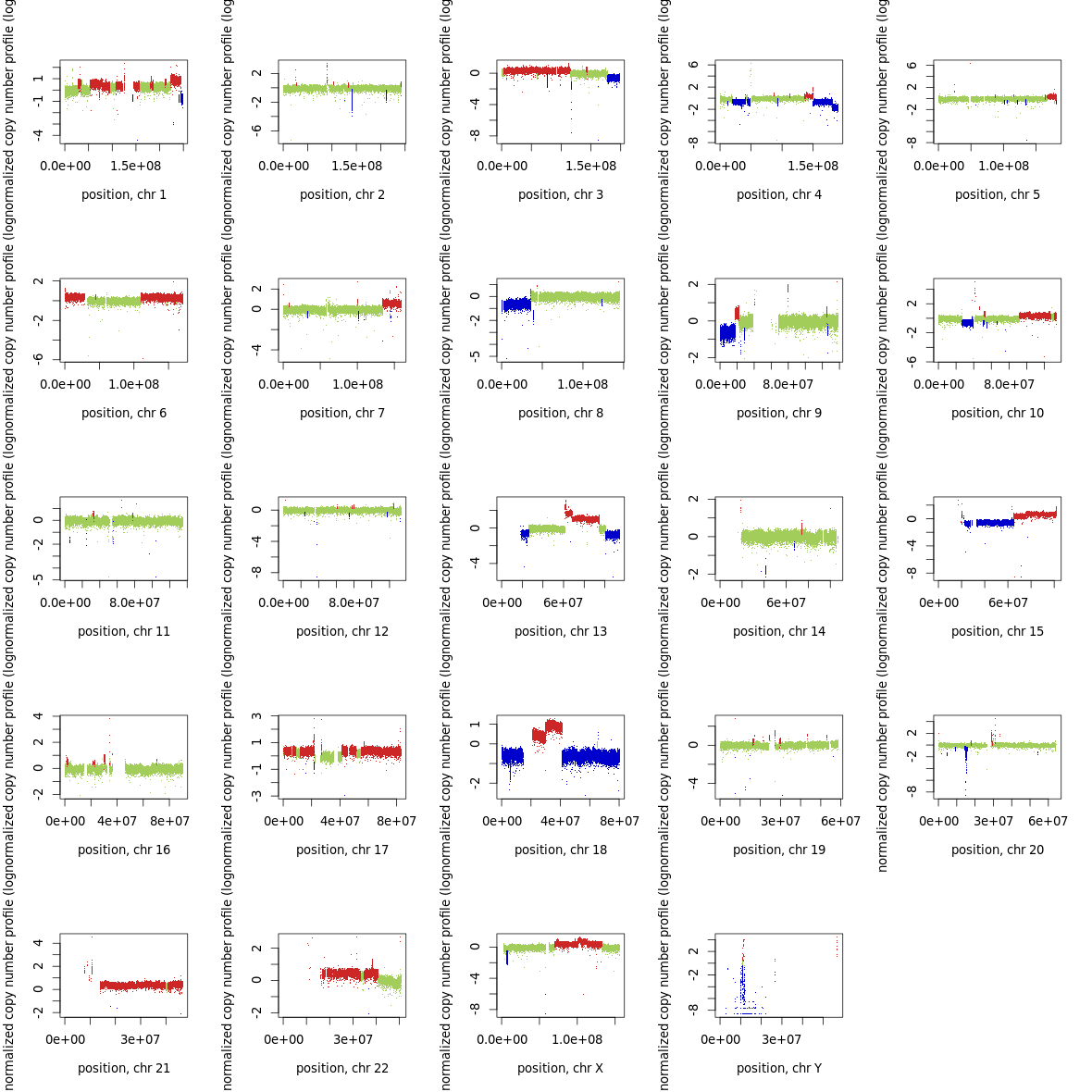
**

**Supplementary Figure 12:** GRK2 expression in WT and PDE4D7 knockdown (P1) LNCaPs via RNAseq (A) and western blot (B).

**Supplementary Table 6: Gene Set Enrichment Analysis (www.gsea-msigdb.org) of Hallmark Pathways (3).** Top 20 Hallmark Pathways depleted in Tet/doxycycline inducible LNCaP knockdown cell line P1 (+ doxycycline) vs Tet inducible LNCaP cell line P1 (- doxycycline) after GSEA. **SIZE** – number of genes in the Hallmark Pathway; **ES** – enrichment score; **NES** – normalized enrichment score; **NOM p-value** – nominal p-value; **FDR q-value** – false discovery rate q-value.

| **NAME** | **SIZE** | **ES** | **NES** | **NOM p-value** | **FDR q-value** |
| --- | --- | --- | --- | --- | --- |
| HALLMARK_TNFA_SIGNALING_VIA_NFKB | 199 | -0.52 | -1.77 | <1.00E-03 | 6.00E-03 |
| HALLMARK_HYPOXIA | 200 | -0.51 | -1.75 | <1.00E-03 | 4.00E-03 |
| HALLMARK_MYC_TARGETS_V2 | 58 | -0.6 | -1.72 | 2.00E-03 | 3.00E-03 |
| HALLMARK_EPITHELIAL_MESENCHYMAL_TRANSITION | 200 | -0.45 | -1.53 | <1.00E-03 | 2.80E-02 |
| HALLMARK_MYC_TARGETS_V1 | 200 | -0.44 | -1.51 | <1.00E-03 | 2.90E-02 |
| HALLMARK_UV_RESPONSE_DN | 144 | -0.44 | -1.45 | 7.00E-03 | 4.60E-02 |
| HALLMARK_GLYCOLYSIS | 200 | -0.42 | -1.44 | <1.00E-03 | 4.20E-02 |
| HALLMARK_ESTROGEN_RESPONSE_EARLY | 200 | -0.42 | -1.41 | 2.00E-03 | 4.70E-02 |
| HALLMARK_NOTCH_SIGNALING | 32 | -0.53 | -1.38 | 6.30E-02 | 5.80E-02 |
| HALLMARK_WNT_BETA_CATENIN_SIGNALING | 42 | -0.49 | -1.31 | 8.70E-02 | 1.06E-01 |
| HALLMARK_E2F_TARGETS | 200 | -0.37 | -1.25 | 3.20E-02 | 1.71E-01 |
| HALLMARK_MTORC1_SIGNALING | 200 | -0.37 | -1.24 | 4.40E-02 | 1.82E-01 |
| HALLMARK_ANGIOGENESIS | 36 | -0.46 | -1.2 | 1.52E-01 | 2.24E-01 |
| HALLMARK_ANDROGEN_RESPONSE | 100 | -0.38 | -1.2 | 1.04E-01 | 2.26E-01 |
| HALLMARK_INFLAMMATORY_RESPONSE | 200 | -0.34 | -1.17 | 7.30E-02 | 2.56E-01 |
| HALLMARK_HEDGEHOG_SIGNALING | 36 | -0.43 | -1.14 | 2.36E-01 | 3.16E-01 |
| HALLMARK_APICAL_SURFACE | 44 | -0.42 | -1.14 | 2.22E-01 | 3.11E-01 |
| HALLMARK_IL2_STAT5_SIGNALING | 199 | -0.33 | -1.12 | 1.30E-01 | 3.32E-01 |
| HALLMARK_ESTROGEN_RESPONSE_LATE | 200 | -0.33 | -1.12 | 1.18E-01 | 3.17E-01 |
| HALLMARK_KRAS_SIGNALING_UP | 200 | -0.33 | -1.12 | 1.25E-01 | 3.01E-01 |

**Supplementary Table 7: Primers and probe sequences used for RT-qPCR.** PUM1, TBP, ACTB and HPRT1 were used as housekeeping genes. All probes were labelled 5' 6-FAM/ZEN/3' IBFQ.

| **Gene** | **Accession No.** | **Forward Primer (5’-3’)** | **Reverse Primer (5’-3’)** | **Probe (5’-3’)** |
| --- | --- | --- | --- | --- |
| PDE4D7 | NM_001165899.1 | GAACATTCAACGACCAACCA | TGCCATTGTCCACATCAAAA | CTGCCGCTGATTGCTATCACTTCTGCA |
| PUM1 | NM_001020658.2 | GCCAGCTTGTCTTCAATGAAAT | CAAAGCCAGCTTCTGTTCAAG | ATCCACCATGAGTTGGTAGGCAGC |
| TBP | NM_003194.4 | GCCAAGAAGAAAGTGAACATCAT | ATAGGGATTCCGGGAGTCAT | TCAGAACAACAGCCTGCCACCTTA |
| HPRT1 | NM_000194.2 | GAGGATTTGGAAAGGGTGTTTATT | ACAGAGGGCTACAATGTGATG | ACGTCTTGCTCGAGATGTGATGAAGG |
| TUBA1B | NM_006082.3 | TGACTCCTTCAACACCTTCTTC | TGCCAGTGCGAACTTCAT | CCGGGCTGTGTTTGTAGACTTGGA |

**Supplementary Methods – Additional Details**

**Generation of stable PDE4D7 knockdown LNCaPs**

Both the PDE4D7-targeting shRNA, and the scrambled control transfer plasmid, were transfected with packaging and the envelope plasmids into HEK193T cells for virus production. For transduction of the LNCaP target cell line with lentiviruses, cells were seeded in 6-well plates at 0.2x10^6^ cells per well in 2 ml growth medium. When cells reached ~30-40% confluence they were infected with lentiviruses at MOI=10 in the presence of 10 µg/ml Polybrene. Cells were incubated with lentivirus for ~16 hours (overnight), and then medium changed. 48 hours after infection puromycin was added to the final concentration 2 µg/ml. The culture medium change was done every 3-4 days. After 7 days of treatment with puromycin cells were transferred from 6-well plate to 10 cm dishes for single cell colony selection (also in the presence of puromycin). Selected colonies were transferred into separate wells in 6-well plates. Upon reaching 80% confluence, cells were detached by trypsin and seeded in larger vessels (e.g., 10-cm cell culture dishes) for expansion. RNA from cells was extracted using RNeasy kit (Qiagen). cDNA was synthesized using either oligo-dT or specific primers. qPCR to verify gene knockdown was done using PrimeTime Gene Expression Master Mix (IDT, Cat. 1055772). For all downstream molecular analysis including RNA sequencing and whole genome sequence (WGS) we analyzed the following cell lines/clones: LNCaP FCG wildtype (WT); LNCaP transfected with scrambled shRNA control (clone SC2); LNCaP transfected with PDE4D7 targeting shRNA (clones P1 and w5.2).

**Inducible PDE4D7 re-expression in PDE4D7 knockdown LNCaP P1 cells**

In Tet-On system, the target gene expression is initiated when reverse tetracycline-controlled transactivator (rtTA) is enabled to bind to Tet promoter in the presence of tetracycline or its analogues (doxycycline). VSV-G lentivirus particles were produced using 3rd generation packaging system. Transduction with lentiviruses was done at MOI=5-10 in the presence of 8 µg/ml polybrene. Cells transduced with CMV promoter lentivirus were cultured in 10 cm Petri dishes. Antibiotic treatment with puromycin at concentration 1.5 µg/ml was done for 7 days. Several colonies were selected for individual expansion. Remaining colonies have been pooled.

Cells transduced with Tet-On inducible lentivirus have been tested for the induction of PDE4D7 expression in the presence of 1 µg/ml Doxycycline. Clonal selection was done using limiting dilution method. Pool of cells was diluted to the cell concentration of 8 cells/ml and 0.1 ml aliquots were loaded into several 96-well plates and cultured for two weeks. Plates were monitored daily under microscope and wells containing single cell were marked. Over 10 single cell colonies have been selected for expansion and tested for PDE4D7 expression in the presence of Doxycycline. Expression of PDE4D7 has been estimated by RT-PCR.

**Whole Genome Sequencing**

DNAseq Data Processing: Analysis of the WGS samples was carried out using the Sarek pipeline v. 2.7.1 available for the standardized workflow Nextflow v. 21.10.6. Briefly, sequencing quality was assessed with FastQC v. 0.11.9. Low quality bases appeared to be already trimmed by the provider, as well as any trace of adapter in them, therefore the trimming step was skipped. Cleaned reads were aligned to the reference human genome version GRCh38 with the Burrows Wheeler Aligner bwa-mem v.0.7.17. The Genome Analysis ToolKit (GATK) v. 4.1.7.0 was used for marking duplicated reads and performing Base Quality Score Recalibration. Germline variants in the wildtype and transfected germline were called with Strelka2 v. 2.9.10, while somatic variants were called with Strelka2 and GATK’s Mutect2 algorithm. The effect of germline and somatic variants and their functional annotation was predicted with SnpEff v. 4.3. Structural Variants were identified with Manta v. 1.6.0 and TIDDIT v. 2.7.1, while differences in Copy Number Variants between wildtype and transfected cell lines were predicted with Control-FREEC v. 11.6. Results of the various steps were summarized in an interactive graphical report with MultiQC v.1.12. Additionally, a custom pipeline was implemented for an initial identification of the putative insertion sites of the viral vector in the transfected cell line genome.

Germline, Somatic and Structural variants called with Strelka2, Mutect2 and Manta tools in the wildtype and transfected cell lines were independently used for the identification of putative insertion sites. Called insertions were first filtered by length with a custom python script, using two minimal length thresholds of 50 bp and 100 bp. The nucleotide sequences of the long insertions were then collected and aligned against the nucleotide sequence of the viral vector with NCBI blast+ v.2.12.0. This step allowed the identification of insertions with sequence similarity to the viral vector. Long insertions were proposed as putative insertion sites of the viral vector in the cell line genome if they met the following requirements: i) Having high sequence similarity (>95% percentage identity) to the viral vector sequence; ii) Being absent in the germline variants called for the wildtype cell line genome; iii) Being identified concordantly in the germline analyses of the transfected cell line genome and in the somatic variants called as differences between transfected and wildtype cell line genomes.

**RNA sequencing**

RNAseq Data Processing: The quality of sequencing data (fastq files) was checked using FastQC v.0.11.9, and MultiQC v.1.9. Illumina sequencing adapters and low-quality parts at the 3 prime ends of the reads were removed using SeqPurge v.2021_12. Quality-based trimming of reads, polyA and adapters was performed using Trim Galore! v.0.6.7. Ribosomal RNA derived reads were identified and removed by mapping all reads to a set of rRNA se­quences using the non-gapped mapper bowtie2 v.2.4.5.0 [1]. All remaining reads were mapped to the human reference genome (fasta file of the reference genome, gtf file of the genome annotation, ENSEMBL release 96) using STAR v.2.7.2b [2]. Expression of genes was quantified by counting the reads mapping to any exon of a gene using featureCounts v.2.0.1 with default settings, respecting the strandedness of the sequencing library.

Gene expression TPM calculation: To ensure comparability of expression values between samples all read counts were normalized by the transcripts per million (TPM) method as implemented in the RSEM v1.3.1 algorithm [3].

**Calculation of Clinical and Genomic Risk Scores**

- **Calculation of the clinical risk score CAPRA-S**

The postsurgical CAPRA-S risk score and its corresponding low-risk (1), intermediate-risk (2), and high-risk (3) categories were calculated as described earlier [24]. The EAU-BCR score and its two categories of low and high postsurgical risk of disease progression were determined as published [25].

- **Calculation of the PDE4D7 Score and PDE4D7 Score Categories**

Generation of normalized PDE4D7 transcript expression was performed by subtracting the RT-qPCR Cq of the PDE4D7 transcript from the averaged RT-qPCR Cq of the reference genes and transformed to the PDE4D7 score and its related PDE4D7 score classes [21]. In multivariable Cox and logistic regression analyses for various available biological and treatment-related outcomes, the PDE4D7 score was used as a continuous variable. For Kaplan-Meier survival analysis, we transformed the normalized PDE4D7 transcript expression values to a percentile distribution (pPDE4D7 score), which allowed more efficient comparison with other genomic scores, as all scores were distributed between 0-1 (see also below). Two cut-offs for the pPDE4D7 score were defined by AUROC (area under the ROC curve) analysis with 5-year prostate cancer-specific death after the start of SRT as the dependent variable and the pPDE4D7 score as the independent variable. One cut-off (pPDE4D7>0.2) was defined as the point in the AUROC with maximum sensitivity and specificity. The second cut-off was defined as the point in the AUROC at max sensitivity (pPDE4D7>0.87). Consequently, the pPDE4D7 score classes stratified patients into three sub cohorts (pPDE4D7 scores >0.87: ‘high’; pPDE4D7 scores >0.2 and <=0.87: ‘intermediate’, and pPDE4D7 scores <=0.2: ‘low’).

- **Calculation of Cell Cycle Progression and the Genomic Prostate Score**

To derive the risk scores for two commercially available prognostic tests that use the CCP (Cell Cycle progression) 31 gene signature and GPS (Genomic Prostate Score) 12 gene signature, we inferred the mxCCP and mxGPS scores from RNAseq expression data of the respective signature and reference genes based on previously published formulas to calculate the respective risk score [26]. The derived mxCCP and mxGPS scores were converted to percentile distributions (mxpCCP and mxpGPS, respectively). Where indicated, we either used the mxCCP and mxGPS (AUROC analysis, Cox regression) or the mxpCCP and mxpGPS (Kaplan-Meier survival) for use in downstream statistical analysis.

**Western blotting: List of primary and secondary antibodies**

anti-PDE4D5 1:5000 (Baillie lab), anti-PDE4D7 1:500 (Baillie lab), anti-PDE4D9 1:5000 (Baillie lab), anti-Pan-PDE4D 1:5000 (Baillie lab), anti-VSV 1:5000 (Abcam, #ab1874), anti-GAPDH 1:5000 (Abcam, #ab8245), anti-AR 1:1000 (Cell Signal, #D6F11), anti-PSMA 1:1000 (Invitrogen, #1H8H5), anti-E-cadherin 1:500 (Cell Signaling, #3195S), anti-TMPRSS2 1:1000 (Abcam #ab109131), anti-NKX3.1 1:1000 (Cell Signaling, #92998), anti-PSA 1:1000 (Abcam, #ab76113), anti-AR-V7 (Cell Signaling, #68492), and anti-GR (Cell Signaling, #12041T). The secondary antibodies used were IRDye® 800CW Donkey-anti-Rabbit IgG 1:5000 (LI-COR, #926-32213), IRDye® 680RD Donkey-anti-Goat IgG 1:5000 (LI-COR, #926-68074), IRDye 800CW Goat-anti-Human IgG 1:5000 (LI-COR, #926-32232), and IRDye® 680RD Donkey-anti-Mouse IgG 1:5000 (LI-COR, #925-68072).

**References:**

1. Langmead, B., & Salzberg, S. L. Fast gapped-read alignment with Bowtie 2. Nature methods 2012, 9(4), 357-359. https://doi.org/10.1038/nmeth.1923

2. Dobin, A., Davis, C. A., Schlesinger, F., Drenkow, J. et al. STAR: ultrafast universal RNA-seq aligner. Bioinformatics 2013, 29(1), 15-21. https://doi.org/10.1093/bioinformatics/bts635

3. Li B, Ruotti V, Stewart RM, Thomson JA, Dewey CN. RNA-Seq gene expression estimation with read mapping uncertainty, Bioinformatics 2010, **26**: 493–500. https://doi.org/10.1093/bioinformatics/btp692
